# Variations in health-related quality of life across sociodemographic groups, health conditions, and modifiable risk factors: a population-based EQ-5D-3L study

**DOI:** 10.1186/s12955-026-02587-9

**Published:** 2026-07-20

**Authors:** Mihretab Gebreslassie, Stefan Fors, Per Tynelius, Mathieu F. Janssen, Sofia Sveréus, Emelie Heintz

**Affiliations:** 1 Centre for Epidemiology and Community Medicine, Region Stockholm, Stockholm, Sweden; 2 Department of Global Public Health, Karolinska Institutet, Stockholm, Sweden; 3 Aging Research Centre (ARC), Karolinska Institutet & Stockholm University, Stockholm, Sweden; 4 Medical Psychology and Psychotherapy, Erasmus MC, Rotterdam, The Netherlands; 5 EuroQol Group, Rotterdam, The Netherlands; 6 Stockholm Centre for Health Economics, Region Stockholm, Stockholm, Sweden; 7 Health Economics and Policy Research Group, Department of Learning, Informatics, Management and Ethics, Karolinska Institutet, Stockholm, Sweden

**Keywords:** EQ-5D, Health-related quality of life, Health inequality, Self-reported health, Population health, Sweden

## Abstract

**Background:**

EQ-5D is widely used in economic evaluations and population health assessments. This study used a large population-based cohort with EQ-5D-3L data to describe the health-related quality of life (HRQoL) of the general population in Stockholm County and investigate potential inequalities and variations in HRQoL.

**Methods:**

This study analysed data from 52,714 respondents aged 23–104 years from the 2021 Stockholm Public Health Cohort (SPHC). Calibration weights were used to address non-response bias and improve representativeness. Differences in mean EQ-VAS and EQ-5D-3L index values were analyzed using multivariable regressions, and modified poison regression was used to explore the likelihood of reporting problems across the EQ-5D dimensions. Splines were used to flexibly model the nonlinear relationship between age and HRQoL.

**Results:**

A total of 169 health states were observed, with 37% of the respondents reporting “no problems” in all dimensions (“11111”). Further, 0.05% (*n* = 14) respondents reported the worst possible health state (“33333”). The HRQoL of the general population was generally good but variations across sociodemographic and risk factor groups were observed. HRQoL demonstrated a non-linear relationship with age, with relatively lower scores observed among the youngest adults, increasing through midlife, followed by a marked decline in older age, particularly among those aged 80 years and above. The mean EQ-5D-3 L index was lower among females (0.79 [95% CI: 0.79–0.80]) than males (0.83 [95% CI: 0.83–0.84]), and a similar pattern was observed for the mean EQ-VAS scores (75.96 [95% CI: 75.59–76.33] vs. 78.3 [95% CI: 77.62–78.42], respectively). People with lower education, low disposable income as well as those born outside of the European union and UK reported relatively lower HRQoL. Adjusting for age attenuated but did not eliminate these demographic and socioeconomic differences. Persistent associations between behavioural risk factors and HRQoL were also observed. Similarly, people with chronic health conditions, particularly those with long-term illness or multiple health conditions, exhibited markedly lower HRQoL.

**Conclusion:**

This study demonstrated substantial variations in HRQoL across sociodemographic groups, behavioural risk factors, and self-reported health conditions, providing insights that can inform healthcare planning and future investments in health and research. The results may also serve as reference values in clinical and economic evaluations, and for comparing outcomes from specific patient subgroups with the general population.

**Supplementary Information:**

The online version contains supplementary material available at 10.1186/s12955-026-02587-9.

## Introduction

Contemporary health systems aim to maximize population health, while containing cost and minimizing health inequalities. To this end, in Sweden, as in several other countries, legislation stipulates, among others, that individuals in greater need should be given priority, and limited healthcare resources should be used efficiently [1–3]. One of the fundamental prerequisites for implementing as well as evaluating progress toward these goals, is measuring the general population’s health and identifying subgroups with the highest burden of illness. This requires robust, generic instruments that can capture health status and health-related quality of life (HRQoL) of the general population, as well as of specific patient groups.

The EQ-5D, developed by the EuroQol Group, is one of the most widely used generic preference-weighted instruments for assessing and valuing HRQoL [4]. At present, it is the preferred instrument for health technology assessment (HTA) in numerous contexts [5], and its use has extended beyond economic appraisals to include clinical trials, observational research, population health surveys, and as a patient-reported outcome measure (PROM) in routine clinical practice [6].

EQ-5D data from population surveys can be used to describe self-reported HRQoL of the general population and explore disparities in health. A thorough description and continued assessment of the HRQoL of the general population is essential for understanding the burden of diseases, identifying unmet needs and potential targets for interventions, and healthcare planning [7–10]. This is particularly relevant in light of modern medicine’s growing emphasis on reducing morbidity and enhancing functional outcomes. Also, examining sociodemographic differences in HRQoL can provide nuanced and actionable evidence to identify avoidable health inequalities and thereby support policy development towards increased equity in health. The latter is highly relevant not least given the persistent, and in some cases widening, inequalities in mortality [11–13].

The Stockholm Public Health Cohort (SPHC), an open cohort followed through public health surveys conducted every four years since 2002 for health and risk factor surveillance, contains large population level EQ-5D-3L data which could be leveraged for such purposes. These data have been linked to national registries to obtain detailed sociodemographic information, enabling more robust and granular analysis of social inequalities in health, yet they have been underutilized in research and policy applications. This study utilised this unique source of data to describe the HRQoL of the general population in Stockholm County, investigate potential social inequalities and variations in HRQoL related to common modifiable risk factors and prevalent health problems. Such evidence can support local decision-making within Sweden’s decentralised healthcare system, where regional authorities are responsible for financing and delivering healthcare services [1]. Moreover, this data can also be used to establish population norms [14–16], which can serve as a benchmark against which the outcomes of patients and specific subgroups can be compared, either by themselves or through the use of quality-adjusted life years (QALYs) [17], which are widely used for reimbursement decisions and priority setting in healthcare [9, 18–20].

While Swedish national population norms for the EQ-5D-3L have been published previously [21, 22], these estimates rely on data from more than two decades back, and may have limited contemporary relevance given subsequent sociodemographic and epidemiological changes. There is also published population norms [23] for the more recently developed version of EQ-5D, EQ-5D-5L. However, there remains continued need for an updated EQ-5D-3L data alongside the newer version for several reasons. First, several of the Swedish NQRs that collect EQ-5D data are still using the EQ-5D-3L version [24, 25]. Second, national decisions regarding reimbursement of pharmaceuticals in Sweden are still based on QALY calculations with health state values from the EQ-5D-3L version [5, 26–28]. And third, many health economic models will continue to rely on EQ-5D-3L data reported in the literature and therefore require corresponding population norms from the same instrument to ensure appropriate comparisons. Furthermore, the study by [23] is based on survey data from the mid Sweden Counties (i.e. Sörmland, Uppsala, Värmland, Västmanland and Örebro) and does not include data from Stockholm County.

## Methodology

### Setting, data and participants

Stockholm County is the largest county in Sweden with a total population of about 2.4 million, accounting for over a fifth of the Swedish population. At the time of data collection in 2021 it consisted of 38 administrative municipalities and urban districts, and the great majority of the population is urban. Over a fourth of the population were born outside of Sweden and another fourth from outside Stockholm. Region Stockholm is responsible for both outpatient and inpatient care [1] in Stockholm County – in accordance with the decentralised healthcare system in Sweden.

The data come from the SPHC, which contains a combination of repeated cross-sectional and longitudinal data, linked with national registries. The SPHC samples were based on a cross-sectional samples selected from the Swedish Total Population Register [29, 30]. Self-reported data were collected every 4 years since 2002, and currently the SPHC contains data from 2002, 2006, 2010, 2014 and 2021. The cross-sectional surveys involved area stratified, random samples of the population of Stockholm County aged 18–84 years (2002, 2006) and > = 18 years in 2010 and 2014. A full description of the survey, the questionnaires and technical reports are available from the SPHC study website [31].

In 2021, a follow-up survey, where EQ-5D-3L was also included, was sent out to all the participants who had participated in any of the previous surveys. A total of 66,595 individuals were invited to participate, with the option of responding either via paper questionnaire or online. The overall response rate was 79.2% (*N* = 52,736), with approximately 72% (*N* = 37,964) of them completing the paper version and the remaining 28% (*N* = 14,772) responding via the web-based version.

### Handlings of missing data and representativeness

To deal with bias due to non-participation in the 2021 follow-up, we derived calibration weights based on the same method [32] that had been used in the previous cross-sectional surveys (2021 technical report [31]). The calibration weights were designed to ensure that the estimates more accurately reflect the overall population of Stockholm County. They were derived from national register data covering the entire Stockholm population and were based on the following variables: age, sex, country of birth, civil status, educational level, and area of residence. All analyses applied survey analysis procedures to account for theses calibration weights. Item-level missingness was generally low (Table 1). Given the minimal extent of missing data, complete-case analyses were conducted using only those with complete data on all sociodemographic factors (age, sex, income, education, and country of birth) and either all EQ-5D dimensions or EQ VAS. Available cases were used for additional analysis on variations of HRQoL across behavioural risk factors and prevalent health conditions.

### Measurement of exposure and outcome variables

The EQ-5D-3L data were collected through postal and web-based questionnaires, using the Swedish version of the EQ-5D-3L questionnaire. The EQ-5D comprises a descriptive system, covering five dimensions of health—mobility, self-care, usual activities, pain/discomfort, and anxiety/depression—each with three response levels (no, some, or extreme problems), resulting in 3^5^ = 243 possible health states, and a visual analogue scale (EQ VAS) – in which respondents indicate their subjective state of health on a scale ranging from the “worst” (0) to the “best imaginable health” (100). Health states derived from the EQ-5D responses can be converted into a single index value using national value sets that assign preference weights to all possible EQ-5D states. The health profiles in this study were transformed into index values using the UK value set for EQ-5D-3L [33]. This was chosen over the Swedish experience-based value set [34] which is on another scale than the UK value set [33], since it is the most commonly used value set in HTA in Sweden [26–28, 35]. Briefly, the main difference between these two value sets lies in the perspective of the respondents to the valuation tasks used to construct the value sets. In the UK value set [33], preference weights were generated by asking representatives of the general population to value a description of hypothetical EQ-5D-3L health states, whereas in the Swedish value set [34], also commonly referred to as an “experience-based value set”, respondents in a general population survey were asked to value their own current health state.

Socio-demographic data were retrieved from the Total Population Register [30] and Integrated Database for Labor Market Research (LISA) [36] from Statistics Sweden (SCB). The following variables were included: sex, categorized as male/female; age (categorized into 5-year intervals, and 10-year intervals in the regressions); education, categorized as pre-secondary (primary education), secondary (at least two years of high-school education) and post-secondary education (university or other higher education); country of birth, categorized as *Sweden*, *Nordic*, *EU28* (individuals born in the EU27 countries or UK), and *other (*the rest of the world*)*; income, based on annual equivalised disposable household income, categorized into quintiles using cut-offs from the general population of Stockholm County in 2021 as Q1 (< 182,300), Q2 (182,300 − 263,800), Q3 (263,800 − 345,700), Q4 (345,700 − 462,800) and Q5 (> 462,800 SEK). Participants that had zero or negative disposable household income (0.26%) were included in the lowest quintile.

All behavioural and metabolic risk factors were based on self-reported data. Body mass index (BMI in kg/m2) was calculated as height divided by weight squared and was categorized as underweight (< 18.5), normal weight (18.5–24.9), overweight (25–29.9), obesity class I (30–34.9), obesity class II (35–39.9), and obesity class III (≥ 40). Smoking status was categorised as never (never smoked daily), previous daily smoking or current daily smoking. Sedentary lifestyle was defined by daily sitting time and categorized into < 4 h, 4–9 h, and ≥ 10 h per day. Alcohol consumption was categorized into abstaining, occasional drinking (but below cut-off for hazardous alcohol consumption) and hazardous drinking, based on the Alcohol Use Disorders Identification Test – Consumption (AUDIT–C) questionnaire in line with the Swedish national clinical guidelines [37].

Self-reported general health, based on the question “How is your general health?”, was categorized as *very good*,* quite good*,* neither good nor bad*,* quite bad*,* or very bad*. The long-term illness question “Do you have any long-term illness, health problems following an accident, disability or other chronic health problem?” was dichotomised into a Yes/No. Physician diagnosed health conditions were assessed with the question “Have you been diagnosed by a doctor with any of the following conditions?“, with response alternatives: Diabetes, Chronic Obstructive Pulmonary Disease (COPD), Psoriasis, High blood lipids, Angina pectoris, Heart failure and Asthma. Hypertension was based on the question “Are you currently receiving treatment for high blood pressure?“. Neck pain, low back pain, and headache were assessed using self-reported responses to the following questions: “In the past 6 months, have you had pain in the neck or shoulders?”, “In the past 6 months, have you had pain in the lower back?”, and “In the past 6 months, have you had recurring headaches or migraines?”, respectively. Multimorbidity is operationalised in this study as having multiple health conditions; any combination of the self-reported or physician diagnosed health conditions listed above.

### Data analysis

Descriptive statistics are presented as weighted means with corresponding 95% confidence intervals (CIs) for the EQ-5D-3L index and EQ VAS scores, both for the total population and for subgroups defined by sociodemographic characteristics. For the same groups, the prevalence of reported problems in each EQ-5D dimension is reported as weighted proportions with 95% CIs. To facilitate interpretation and further analysis, the three response levels in each EQ-5D dimension were also dichotomised into “no problems” (level 1) vs. “any problems” (levels 2–3). The cumulative frequencies of reported health profiles and frequency of the worst possible health state are also presented. Such illustrations help reveal patterns of distributions of responses over health profiles [38].

The association between different sociodemographic factors and HRQoL were analyzed to explore potential health inequalities. The generalized linear regression for complex survey data [39] was used to study differences in mean EQ-5D index and EQ VAS scores, whereas a modified Poisson regression model for survey data with a log link and robust standard errors [40] was used to estimate prevalence ratios (PR) of reporting any problems on each EQ-5D-3L dimension, comparing different sociodemographic groups. The models were fitted sequentially, where for each sociodemographic factor (age groups, sex, country of birth, income, and education level), as independent variable, we present unadjusted estimates (Model 0), and estimates adjusted for age using splines (model 1). Natural cubic splines with knots placed at the 5th, 27.5th, 50th, 72.5th, and 95th percentiles of the age distribution were used to capture the non-linear association between HRQoL and age [41].

To examine variations in EQ-5D index and EQ VAS scores across groups defined by behavioural and metabolic risk factors, and self-reported chronic conditions, we employed a similar strategy but extended the models to further adjust for any variation related to sociodemographic differences in line with previous research [23, 42, 43]. That is, first, unadjusted associations for each risk factor and condition (Model 0) were estimated. Next, we adjusted for age using cubic splines (Model 1), and then further adjusted for sex, country of birth, income and education (*Model 2).*

All analyses were conducted in the statistical program R (*R Foundation for Statistical Computing*,* Vienna*,* Austria*) [44]. Statistical significance was based on a 2-tailed significance level of *P* < 0.05.

### Supplementary analyses

We conducted some additional analyses to assess the robustness of our findings. First, as the data used in our study were collected during the COVID-19 pandemic, we considered the possibility that self-reported health problems may be overestimated due to direct infection or broader pandemic-related effects. In an attempt to assess potential biases related to the timing of the data collection, we compared the age and sex specific estimates in our study to two other sources of data from before the pandemic:

1. We compared the EQ-5D index values, EQ VAS scores and levels of impairment across each dimension with individual person level data from the SPHC 2014 wave – cross sectional representative data of the general adult population in Stockholm County in 2014.
2. We also compared our age and sex specific EQ VAS estimates to estimates in Teni et al. 2022 [23]. The study used EQ-5D-5L data which were based on survey data from the mid-Sweden counties of Sörmland, Uppsala, Värmland, Västmanland, and Örebro from 2017; thus, comparisons were only possible to the EQ VAS estimates.

Second, because OLS regression ignores the bounded and skewed nature of EQ-5D index, it can yield biased estimates. To test if similar patterns are observed with alternative modelling approaches, we used a two-part model where we first fitted a logistic regression to estimate the likelihood of reporting less than perfect health and in the next step employed linear regression to estimate differences in index values in the subset with less than perfect health (EQ-5D index less than 1).

Finally, we also conducted additional analysis to demonstrate the differences between estimates based on the UK value set and the experience based Swedish value set. This was mainly to highlight the resulting differences when different values sets are used to convert health states into the EQ-5D index value.

### Ethical aspects

The study was conducted in accordance with the principles of the Declaration of Helsinki [45], and approval was obtained from the Swedish Ethical Review Authority (Dnr: 2024-05350-01).

## Results

### Participant and data characteristics

The sociodemographic characteristics, prevalence of selected diseases and risk factors, and proportion of missing data in the sample are presented in Table 1. The analytical sample included a total of 52,714 observations. The mean age was 52 years, about 51% of the sample were female, and 70% were born in Sweden.

**Table 1:** Participant characteristics for the entire sample, and stratified by sex

Variables	Overall (*n* = 52 714)	Male (*n* = 22 397)	Female (*n* = 30 317)
**Female**,** %**	51.4	--	--
**Age**,** mean (SD)**	51.98 (17.87)	51.61 (17.35)	52.33 (18.34)
**Age**,** n**^**1**^ **(%)**			
23–29	688 (9.7)	262 (9.2)	426 (10.1)
30–34	1 246 (13.2)	436 (13.3)	810 (13.0)
35–39	1 843 (6.4)	679 (6.5)	1 164 (6.3)
40–44	3 045 (8.9)	1 129 (9.2)	1 916 (8.7)
45–49	4 064 (11.7)	1 598 (12.7)	2 466 (10.8)
50–54	4 970 (7.0)	1 993 (6.9)	2 977 (7.1)
55–59	5 585 (7.8)	2 300 (7.9)	3 285 (7.6)
60–64	5 533 (8.7)	2 379 (9.0)	3 154 (8.4)
65–69	5 688 (6.1)	2 554 (6.2)	3 134 (6.0)
70–74	6 492 (6.6)	2 845 (6.3)	3 647 (6.8)
75–79	6 849 (6.8)	3 167 (6.7)	3 682 (7.0)
80–84	4 076 (4.0)	1 952 (3.8)	2 124 (4.2)
85–89	1 752 (2.0)	784 (1.7)	968 (2.4)
90+	883 (1.2)	319 (0.7)	564 (1.6)
**Income quintile**,** n (%)**			
Q1 (poorest 20%)	5 368 (15.3)	1 723 (12.7)	3 645 (17.7)
Q2	10 760 (22.4)	4 124 (21.0)	6 636 (23.8)
Q3	11 080 (23.3)	4 711 (23.4)	6 369 (23.3)
Q4	12 374 (22.0)	5 600 (23.8)	6 774 (20.2)
Q5 (richest 20%)	13 114 (17.0)	6 233 (19.1)	6 881 (15.0)
* % missing*	0.1	< 0.1	0.1
**Education**,** n (%)**			
Pre-secondary	4 947 (17.8)	2 413 (18.3)	2 534 (17.4)
Secondary	18 834 (33.7)	8 405 (34.1)	10 429 (33.4)
Post-secondary	28 818 (48.4)	11 523 (47.6)	17 295 (49.2)
* % missing*	0.7	0.8	0.7
**Country of birth**,** n (%)**			
Sweden	45 450 (70.0)	19 427 (72.0)	26 023 (68.1)
Nordic	2 498 (5.9)	783 (4.3)	1 715 (7.5)
EU28	1 645 (4.4)	747 (4.4)	898 (4.4)
Other	3 120 (19.6)	1 440 (19.3)	1 680 (19.9)
* % missing*	< 0.1	0.0	< 0.1
**BMI groups**,** n (%)**			
Underweight (< 18.5)	711 (1.6)	116 (0.9)	595 (2.3)
Normal weight (18.5–24.9)	25 074 (48.4)	9 308 (43.1)	15 766 (53.4)
Overweight (25.0–29.9)	18 728 (35.1)	9 551 (41.0)	9 177 (29.6)
Obesity Class I (30.0–34.9)	5 591 (11.2)	2 523 (11.9)	3 068 (10.5)
Obesity Class II (35.0–39.9)	1 307 (2.7)	453 (2.2)	854 (3.1)
Obesity Class III (≥ 40.0)	479 (1.0)	164 (0.9)	315 (1.1)
* % missing*	1.5	1.3	1.7
**Smoking status**,** n (%)**			
Daily smoking	2 752 (6.1)	1 032 (5.7)	1 720 (6.5)
Quit	15 332 (22.3)	6 243 (21.0)	9 089 (23.5)
Never	34 194 (71.6)	14 945 (73.3)	19 249 (69.9)
*% missing*	0.9	1.0	0.9
**Alcohol consumption**,** n (%)**			
Never	5 932 (16.6)	2 142 (13.6)	3 790 (19.4)
Not hazardous	38 284 (67.5)	16 154 (68.2)	22 130 (66.9)
Hazardous	8 031 (15.9)	3 902 (18.2)	4 129 (13.7)
* % missing*	0.8	0.8	0.8
**Sitting**,** n (%)**			
<4 h per day	7 762 (14.9)	3 051 (13.5)	4 711 (16.2)
04–09 h per day	34 387 (60.2)	14 440 (58.1)	19 947 (62.1)
10 + hours per day	10 108 (25.0)	4 748 (28.4)	5 360 (21.7)
* % missing*	0.9	0.9	1.0
**Self-reported conditions**,** n (%)**			
chronic neck pain	8 625 (17.2)	2 606 (12.3)	6 019 (21.7)
* % missing*	0.8	0.7	0.9
Chronic low back pain	9 245 (15.9)	3 195 (12.8)	6 050 (18.8)
* % missing*	0.8	0.7	0.8
Chronic headache/migraine	2 699 (6.4)	658 (3.6)	2 041 (9.1)
* % missing*	0.5	0.4	0.6
**Physician diagnosed conditions**,** n (%)**			
Diabetes	4 521 (7.2)	2 505 (8.5)	2 016 (6.0)
* % missing*	1.2	1.0	1.3
Angina pectoris	1 930 (3.0)	1 189 (4.0)	741 (2.0)
* % missing*	2.1	1.9	2.3
Heart failure	2 466 (3.4)	1 400 (4.0)	1 066 (2.8)
* % missing*	2.0	1.8	2.2
Hypertension	18 181 (23.0)	8 588 (23.7)	9 593 (22.3)
* % missing*	0.3	0.3	0.4
Asthma	4 746 (9.7)	1 580 (7.9)	3 166 (11.5)
* % missing*	1.7	1.7	1.7
COPD	1 622 (2.4)	662 (2.3)	960 (2.6)
* % missing*	1.9	1.7	2.0
Psoriasis	2 267 (3.7)	1 012 (4.0)	1 255 (3.5)
* % missing*	2.1	2.0	2.2
Hyperlipidemia	8 414 (11.0)	4 196 (12.2)	4 218 (9.9)
* % missing*	2.0	1.7	2.2
**Long-term illness**,** n (%)**	20 683 (34.3)	8 590 (32.4)	12 093 (36.1)
* % missing*	0.7	0.5	0.8
**Multimorbidity**^**2**^, **n (%)**			
0	20 506 (48.0)	8 838 (51.5)	11 668 (44.6)
1	14 427 (24.9)	6 002 (23.9)	8 425 (25.7)
2	9 094 (14.3)	3 783 (12.6)	5 311 (15.8)
3	4 933 (7.3)	2 125 (6.7)	2 808 (7.9)
4+	3 726 (5.6)	1 637 (5.2)	2 089 (5.9)
* % missing*	< 0.1	< 0.1	< 0.1
**Self-rated health**,** n (%)**			
Very good	11 504 (23.6)	5 072 (25.5)	6 432 (21.9)
Good	25 935 (49.4)	11 304 (50.5)	14 631 (48.3)
Neither	12 483 (21.9)	4 910 (19.5)	7 573 (24.2)
Bad	2 273 (4.3)	897 (3.8)	1 376 (4.8)
Very bad	342 (0.8)	138 (0.7)	204 (0.9)
* % missing*	0.3	0.2	0.3
**EQ-5D-3 L index**,** mean (SD)**	0.81 (0.22)	0.83 (0.20)	0.79 (0.23)
* % missing*	2.5	2.5	2.5
**EQ VAS**,** mean (SD)**	76.91 (16.69)	77.95 (15.97)	75.92 (17.29)
* % missing*	2.5	1.9	3.0

^*1*^ *N is unweighted number of observations and % is weighted proportion*

^*2*^ *Number of health conditions;Abreviations: SRH, self-rated health; COPD, chronic plumounary disease*

**Fig. 1:**
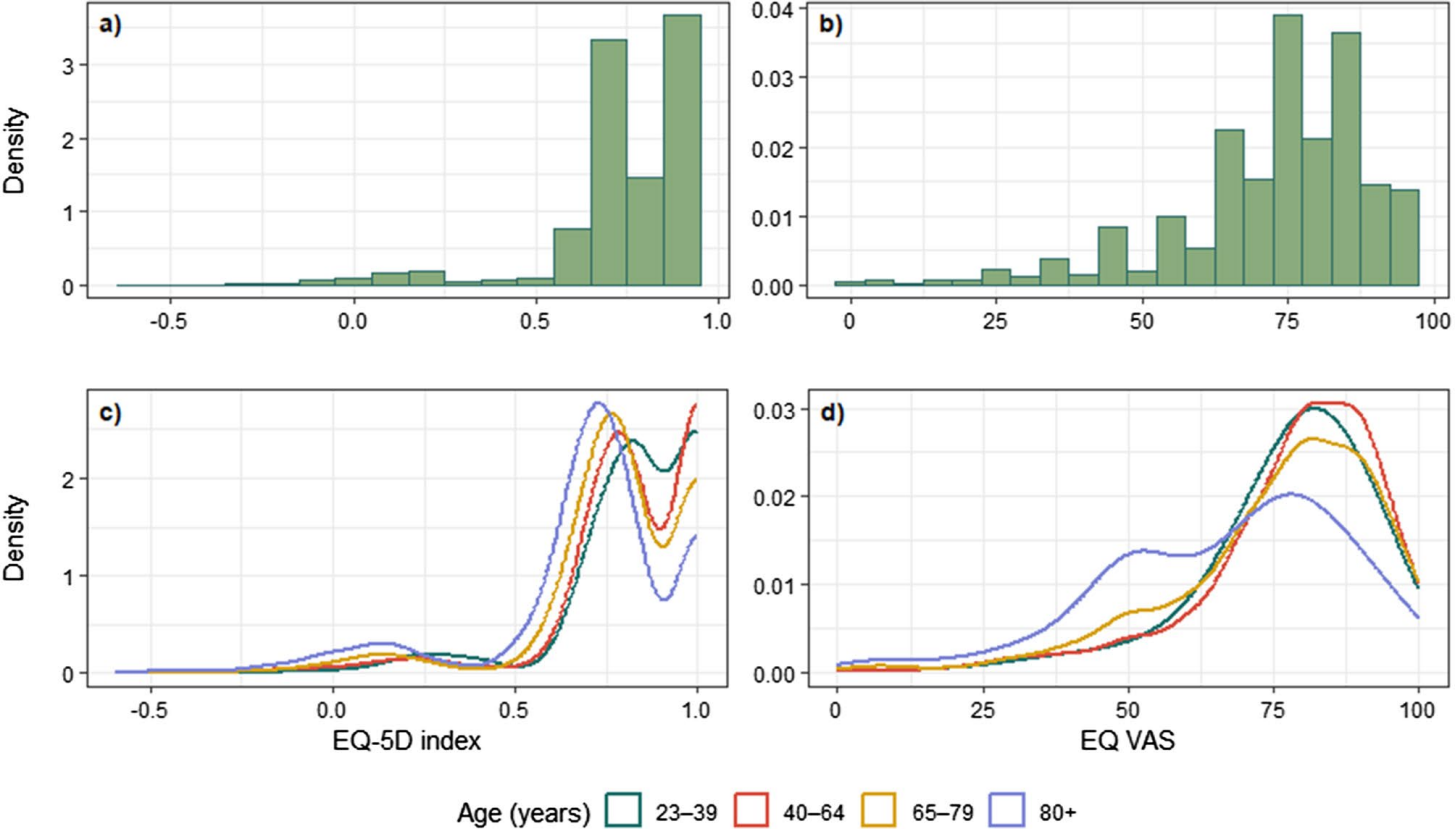
Survey weighted distribution of the EQ-5D-3L index values **(a)** and EQ VAS scores **(b)** for the total sample and stratified by age groups for the EQ-5D-3L index values **(c)** and EQ VAS scores **(d)**

The distribution of the EQ-5D index values (Fig. 1a**)** and the EQ VAS scores (Fig. 1b**)** were both as expected heavily skewed to the left, indicating that majority of the respondents report no problems on the EQ-5D-3L dimensions. The observed EQ-5D index ranged from − 0.59 to 1.0, and EQ VAS score ranged between 0 and 100. However, there was a clear pattern by age, as illustrated in Fig. 1c and d for the EQ-5D index scores and EQ VAS, respectively. The age-stratified panels highlight shifts in HRQoL distributions across the life course, with older age groups generally exhibiting lower index values and EQ VAS scores, particularly the 80 + year olds.

#### Frequently reported health states

A total of 169 health states were observed. In Table 2, the 20 most frequently reported health states, their corresponding EQ-5D-3L index values, the mean EQ VAS scores, and the prevalence of the worst health state (“33333”) are presented. The 20 most frequently reported health states accounted for about 95% of all observations. The best health state (“11111”), i.e. respondents reporting “no problems” in all dimensions, accounted for about 37% of all observations. Health states representing moderate levels of pain/discomfort (11121), and anxiety/depression (11112) were the next most frequently reported. The worst possible health state (“33333”) accounted for 0.05% of all reported health states.

**Table 2:** The survey weighted prevalence of the most frequently observed health states and frequency of reporting of the worst possible health state in EQ-5D-3L, and corresponding index value and mean EQ VAS scores

Health state	Frequency (unweighted *n*)	%	Cumulative %	EQ-5D index value	Mean EQ VAS (se)
11,111	18 222	36.6	36.6	1	86.54 (0.14)
11,121	10 921	16.6	53.2	0.796	80.32 (0.2)
11,112	4361	13.3	66.5	0.848	78.06 (0.31)
11,122	5615	12.0	78.5	0.725	73.11 (0.29)
21,121	2959	3.78	82.3	0.727	72.67 (0.44)
21,122	1628	2.69	84.9	0.656	65.14 (0.65)
21,222	1166	1.97	86.9	0.62	54.54 (0.73)
11,222	702	1.62	88.5	0.689	59.6 (1.16)
21,221	939	1.30	89.8	0.691	61.67 (0.8)
11,123	222	0.78	90.6	0.291	50.61 (1.73)
11,221	379	0.59	91.2	0.76	71.03 (1.00)
21,232	334	0.58	91.8	0.088	47.37 (1.39)
22,222	314	0.58	92.4	0.516	50.19 (1.43)
11,212	169	0.57	92.9	0.812	67.49 (1.44)
11,113	128	0.51	93.4	0.414	58.54 (2.64)
11,223	141	0.50	94.0	0.255	43.48 (2.61)
21,111	327	0.50	94.4	0.85	77.88 (1.58)
11,213	49	0.29	94.7	0.378	51.24 (3.58)
22,232	141	0.28	95.0	-0.016	37.1 (1.97)
22,221	191	0.25	95.3	0.587	53.98 (1.9)
	--	--	--	--	--
33,333	14	0.05	98.8	-0.594	12.03 (8.94)

Cum_prop = cumulative proportion ; se = standard error ; Health states are ordered based on survey weighted %

### HRQoL by sociodemographic subgroups

**Fig. 2:**
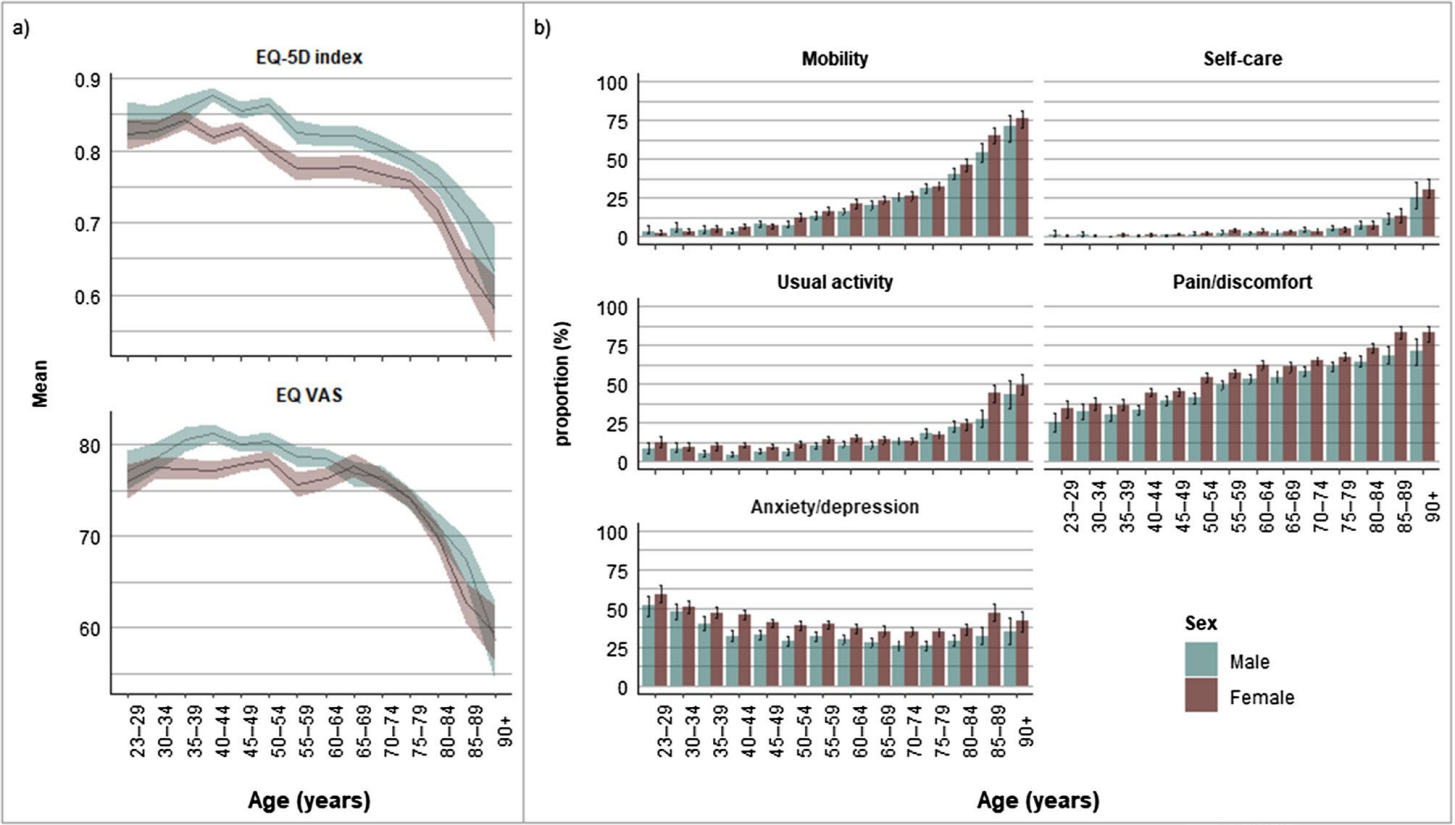
Weighted mean and 95% CI EQ-5D-3L index and EQ VAS by sex and age (**a**), and age- and sex specific prevalence of reporting any problem across EQ-5D-3L dimensions (**b**)

The age and sex stratified mean EQ VAS and EQ-5D index values are presented in Fig. 2a, and values across all sociodemographic characteristics are shown in Table 3. The mean EQ-5D-3L index and EQ VAS scores were generally lower for female respondents (0.79; 95% CI: 0.79–0.80 and 75.96; 95% CI: 75.59–76.33, respectively) compared to male respondents (0.83; 95% CI: 0.83–0.84 and 78.02; 95% CI: 77.62–78.42, respectively). The results also show that HRQoL declines with increasing age, albeit not linearly. The mean EQ-5D-3L index was highest among males aged 40–49 (0.86, 95% CI: 0.86– 0.87), and lowest among females aged 90+ (0.58, 95% CI: 0.53– 0.63). People with lower education, those with low disposable income as well as those born outside of Sweden had generally lower HRQoL (*Age adjusted comparisons are provided in sub-section 3.2.1*).

The age and sex stratified prevalence of some/moderate or severe problems (that is, level 2 or 3) for EQ-5D dimensions are presented in Fig. 2b (*prevalence by other sociodemographic factors are provided in the supplementary* *Table **S1**and proportions for each level of impairment are presented in* *Table **S2*, *in the Additional* *file 1* *in the supplementary*). Generally, it is more common to report problems on the pain/discomfort (52% of female and 43% of male respondents) and anxiety/depression (43% of females and 35% of males) dimensions, and less common on the self-care dimension (3% of females and 2% of males).

The pattern, however, varied depending on the EQ-5D dimension and across age groups (Fig. 2B and supplementary Table S1 & S2, in the Additional file 1). The highest proportion of individuals reporting any problems occurred in the pain/discomfort dimension among those aged 90 years and older (79.6%), whereas the lowest proportion was observed in the self-care dimension among individuals aged 35–39 years (0.75%). Across all EQ-5D dimensions except anxiety/depression, the prevalence of reported problems was higher among older age groups. Anxiety/depression, on the other hand, showed a characteristic bathtub-shaped age distribution. That is, the prevalence was highest among the youngest respondents (highest around 23–29 years: 55.6%), declined to its lowest level around retirement age (lowest in 75–79 years: 30.4%), and then rose again among the oldest old (highest around 85–89 years: 41%). It is also worth mentioning that 29.3% of the population aged 90 years and older reported problems with self-care, compared to 12.6% in those 85–89-year-olds, indicating a sharp decline in HRQoL.

**Table 3:** Weighted means and 95% CI for EQ-5D-3L index values and EQ VAS scores across sociodemographic variables (adult population, 23–104 years old)

	EQ-5D index			EQ VAS		
	All (N* = 51 051)	Male (*N* = 21 648)	Female (*N* = 29 403)	All (*N* = 51 247)	Male (*N* = 21 882)	Female (*N* = 29 365)
**Total**	0.81 (0.81– 0.82)	0.83 (0.83– 0.84)	0.79 (0.79– 0.80)	76.97 (76.69, 77.24)	78.02 (77.62– 78.42)	75.96 (75.59– 76.33)
**Age**						
23–29	0.83 (0.81– 0.85)	0.84 (0.81– 0.87)	0.82 (0.80– 0.85)	76.49 (75.09– 77.89)	77.16 (75.02– 79.31)	75.91 (74.08– 77.74)
30–34	0.83 (0.82– 0.85)	0.84 (0.82– 0.86)	0.83 (0.81– 0.84)	77.98 (76.98– 78.97)	78.49 (76.87– 80.10)	77.49 (76.32– 78.65)
35–39	0.85 (0.84– 0.86)	0.86 (0.84– 0.88)	0.84 (0.83– 0.86)	78.87 (77.94– 79.81)	80.50 (79.13– 81.88)	77.27 (76.03– 78.51)
40–44	0.85 (0.84– 0.86)	0.88 (0.87– 0.89)	0.82 (0.81– 0.83)	79.18 (78.50– 79.85)	81.21 (80.32– 82.10)	77.14 (76.16– 78.13)
45–49	0.84 (0.84– 0.85)	0.86 (0.84– 0.87)	0.83 (0.82– 0.84)	78.98 (78.38– 79.59)	80.05 (79.22– 80.89)	77.79 (76.92– 78.66)
50–54	0.83 (0.82– 0.84)	0.86 (0.85– 0.88)	0.80 (0.79– 0.82)	79.32 (78.61– 80.03)	80.35 (79.31– 81.39)	78.35 (77.38– 79.32)
55–59	0.80 (0.79– 0.81)	0.83 (0.81– 0.84)	0.77 (0.76– 0.79)	77.11 (76.27– 77.96)	78.64 (77.54– 79.74)	75.60 (74.32– 76.88)
60–64	0.80 (0.79– 0.81)	0.82 (0.81– 0.83)	0.78 (0.76– 0.79)	77.41 (76.64– 78.18)	78.51 (77.49– 79.52)	76.30 (75.15– 77.44)
65–69	0.80 (0.79– 0.81)	0.82 (0.81– 0.84)	0.78 (0.76– 0.79)	77.26 (76.33– 78.19)	76.81 (75.44– 78.18)	77.70 (76.44– 78.95)
70–74	0.79 (0.77– 0.80)	0.81 (0.79– 0.82)	0.77 (0.75– 0.78)	76.25 (75.44– 77.06)	76.38 (75.22– 77.54)	76.13 (75.00– 77.27)
75–79	0.77 (0.76– 0.78)	0.79 (0.77– 0.80)	0.76 (0.74– 0.77)	74.04 (73.27– 74.81)	74.07 (72.91– 75.22)	74.02 (73.00– 75.04)
80–84	0.74 (0.72– 0.75)	0.76 (0.74– 0.78)	0.72 (0.69– 0.74)	70.45 (69.35– 71.55)	71.09 (69.58– 72.60)	69.87 (68.30– 71.45)
85–89	0.67 (0.64– 0.69)	0.71 (0.68– 0.74)	0.64 (0.61– 0.67)	64.68 (63.01– 66.34)	67.47 (65.02– 69.93)	62.73 (60.52– 64.95)
90+	0.60 (0.56– 0.63)	0.63 (0.57– 0.69)	0.58 (0.53– 0.63)	59.08 (56.59– 61.56)	58.47 (54.22– 62.71)	59.33 (56.28– 62.38)
**Income**						
poorest 20%	0.73 (0.72– 0.74)	0.75 (0.73– 0.77)	0.71 (0.70– 0.73)	70.41 (69.49– 71.33)	71.88 (70.37– 73.38)	69.41 (68.25– 70.56)
Q2	0.78 (0.77– 0.79)	0.80 (0.79– 0.81)	0.76 (0.75– 0.77)	74.23 (73.61– 74.86)	75.04 (74.07– 76.01)	73.56 (72.76– 74.36)
Q3	0.83 (0.82– 0.83)	0.84 (0.83– 0.85)	0.81 (0.81– 0.82)	77.83 (77.26– 78.40)	78.35 (77.47– 79.24)	77.33 (76.60– 78.06)
Q4	0.85 (0.84– 0.85)	0.86 (0.85– 0.87)	0.83 (0.83– 0.84)	79.96 (79.51– 80.40)	80.70 (80.07– 81.32)	79.13 (78.50– 79.76)
richest 20%	0.86 (0.85– 0.87)	0.87 (0.86– 0.88)	0.85 (0.84– 0.85)	80.97 (80.51– 81.43)	81.30 (80.62– 81.97)	80.57 (79.97– 81.17)
**Education**						
Pre-secondary	0.75 (0.74– 0.76)	0.78 (0.77– 0.79)	0.72 (0.71– 0.74)	72.64 (71.93– 73.34)	73.75 (72.75– 74.75)	71.51 (70.51– 72.51)
Secondary	0.80 (0.79– 0.81)	0.82 (0.81– 0.83)	0.78 (0.77– 0.79)	76.43 (75.95– 76.92)	78.01 (77.30– 78.71)	74.89 (74.24– 75.55)
Post-secondary	0.84 (0.84– 0.85)	0.86 (0.85– 0.87)	0.83 (0.82– 0.83)	78.85 (78.49– 79.21)	79.59 (79.04– 80.14)	78.17 (77.70– 78.64)
**Country of birth**						
Sweden	0.82 (0.82– 0.82)	0.84 (0.83– 0.84)	0.80 (0.80– 0.81)	77.29 (76.99– 77.60)	78.17 (77.71– 78.63)	76.41 (76.01– 76.82)
Nordic	0.77 (0.76– 0.78)	0.79 (0.77– 0.81)	0.76 (0.74– 0.78)	74.37 (73.39– 75.35)	74.47 (72.87– 76.06)	74.31 (73.07– 75.55)
EU28	0.80 (0.78– 0.81)	0.82 (0.80– 0.84)	0.78 (0.76– 0.80)	77.37 (76.21– 78.53)	79.12 (77.61– 80.64)	75.72 (74.02– 77.41)
Other	0.80 (0.79– 0.81)	0.82 (0.81– 0.84)	0.78 (0.76– 0.79)	76.45 (75.69– 77.21)	77.97 (76.89– 79.05)	75.05 (73.99– 76.11)

* N is unweighted count; EU28, European union including UK

#### Results from regression models

**Fig. 3:**
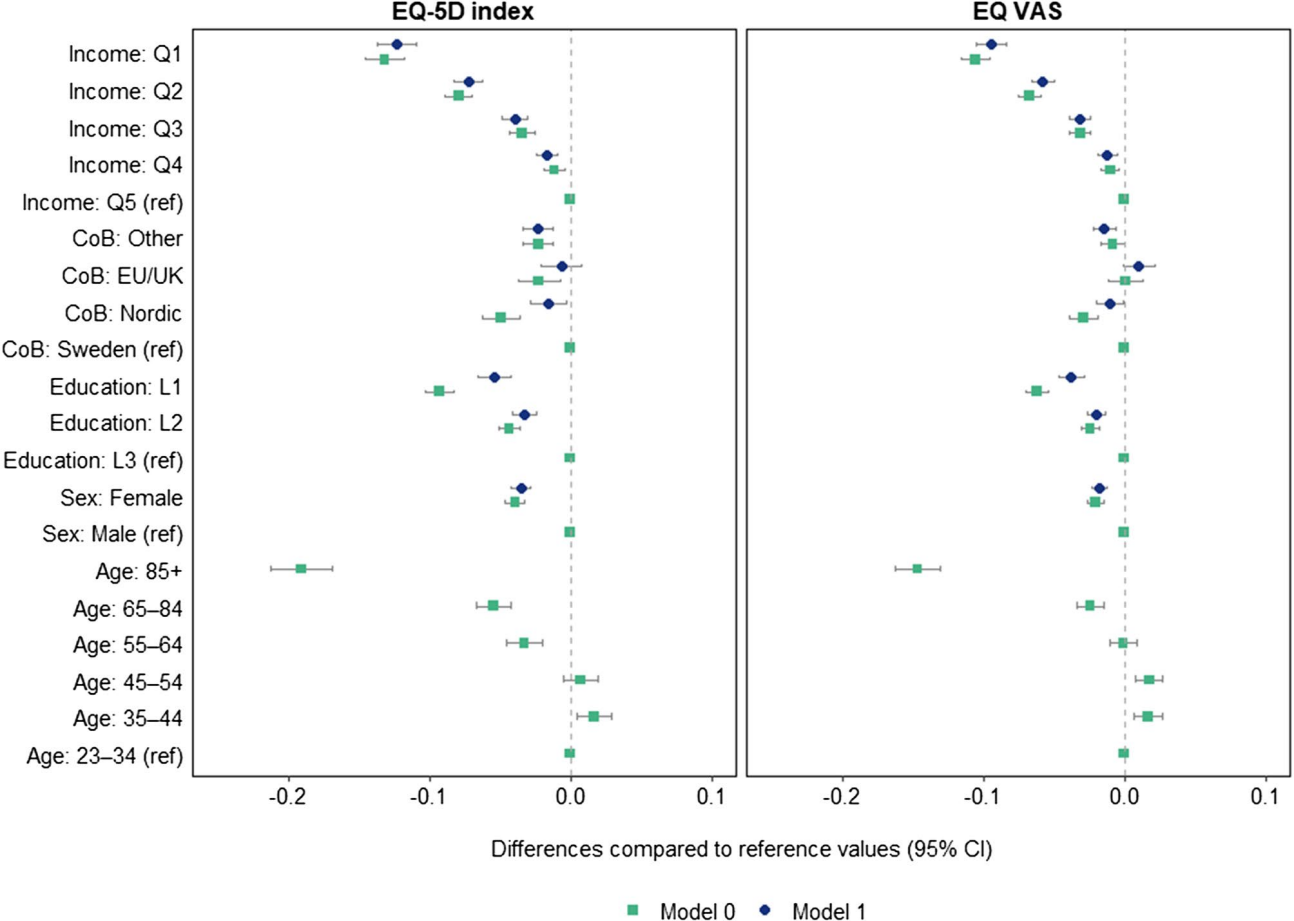
Coefficients and 95% CI across different sociodemographic factors. NB: Coefficients for EQ VAS were estimated using EQ VAS/100 to facilitate comparability with EQ-5D index coefficients. (*Model 0*,* unadjusted coefficients*; *Model 1*,* adjusted for age)*

The EQ-5D index values were lower for female compared to male respondents, and the difference remained even after adjusting for age (Fig. 3). The figure also shows the nonlinear trend of index values across age groups. While a general decline with advancing age was observed, respondents aged 35–44 reported higher values than those aged 23–34. A clearer gradient emerged after age 35, with substantially lower values observed in the oldest age group (85 + years).

Lower socioeconomic status was also associated with poorer HRQoL across different SES measures (Fig. 3). A clear pattern indicating a socioeconomic gradient in HRQoL across educational levels and income groups can be observed, with people with university or higher education and those with higher income reporting statistically significantly higher EQ-5D values. It was also observed that people born outside of Sweden report poorer HRQoL than those born in Sweden (Fig. 3**)**. After adjusting for age, people born outside of Europe (the EU and UK) reported relatively lower EQ-5D index values compared to those born in Sweden. The regression results in Fig. 3 also show that the differences in EQ VAS across sociodemographic groups mirrored the results from the EQ-5D index (*the coefficients of the models for all variables are also provided on the supplementary* *Table **S5*, *Additional* *file **1**).*

Coefficients from Poisson regressions comparing the prevalence of reported problems in EQ-5D-3L dimensions across sociodemographic variables are presented in Table 4. Female sex was associated with reporting higher levels of problems across all dimensions, but the PRs were generally small, ranging from 1.07 (95% CI: 0.89– 1.27) in the self-care dimension to 1.32 (95% CI: 1.21– 1.45) in the usual activities dimension (Table 4). The proportion of people reporting problems increased with increasing age, except on the anxiety/depression dimension (where the prevalence decreased with age). The age gradient was most pronounced for the self-care and mobility dimensions, with PRs of 20.8 (95% CI: 11.3–38.5) and 16.0 (95% CI: 12.4–20.7), respectively, for individuals aged 85 years and older compared with those aged 23–34 years.

**Table 4:** PR and 95% CI of reporting problems across EQ-5D-3L dimensions

	Mobility		Self-care		Usual activities		Pain/discomfort		Anxiety/depression	
	Model 0	Model 1	Model 0	Model 1	Model 0	Model 1	Model 0	Model 1	Model 0	Model 1
**Age**										
23–34	ref	--	ref	--	ref	--	ref	--	ref	--
35–44	1.25 (0.93– 1.69)	--	0.90 (0.44– 1.85)	--	0.78 (0.64– 0.96)	--	1.12 (1.03– 1.23)	--	0.79 (0.74– 0.85)	--
45–54	2.09 (1.58– 2.75)	--	1.75 (0.91– 3.37)	--	0.86 (0.71– 1.04)	--	1.35 (1.25– 1.47)	--	0.68 (0.64– 0.73)	--
55–64	4.18 (3.22– 5.44)	--	3.52 (1.87– 6.64)	--	1.29 (1.08– 1.56)	--	1.70 (1.57– 1.84)	--	0.66 (0.62– 0.71)	--
65–84	7.25 (5.61– 9.36)	--	5.26 (2.86– 9.68)	--	1.65 (1.39– 1.95)	--	1.93 (1.79– 2.08)	--	0.60 (0.56– 0.64)	--
85+	16.0 (12.4– 20.7)	--	20.8 (11.3– 38.5)	--	4.31 (3.60– 5.16)	--	2.40 (2.22– 2.61)	--	0.78 (0.71– 0.86)	--
**Sex**										
Male	ref	ref	ref	ref	ref	ref	ref	ref	ref	ref
Female	1.20 (1.12– 1.28)	1.11 (1.05– 1.19)	1.22 (1.02– 1.46)	1.07 (0.89– 1.27)	1.40 (1.28– 1.53)	1.32 (1.21– 1.45)	1.20 (1.16– 1.24)	1.19 (1.15– 1.23)	1.23 (1.18– 1.29)	1.23 (1.18– 1.28)
**Income**										
Q1 (poorest 20%)	3.05 (2.72– 3.41)	2.40 (2.15– 2.68)	7.70 (5.68– 10.4)	6.20 (4.45– 8.64)	4.32 (3.73– 4.99)	3.85 (3.31– 4.49)	1.37 (1.29– 1.45)	1.35 (1.28– 1.43)	1.68 (1.57– 1.81)	1.65 (1.53– 1.78)
Q2	2.45 (2.20– 2.73)	1.94 (1.74– 2.16)	4.49 (3.33– 6.04)	3.80 (2.75– 5.26)	2.84 (2.46– 3.27)	2.57 (2.22– 2.99)	1.29 (1.23– 1.36)	1.28 (1.21– 1.35)	1.46 (1.36– 1.56)	1.46 (1.36– 1.56)
Q3	1.52 (1.34– 1.71)	1.66 (1.49– 1.86)	1.91 (1.35– 2.71)	2.17 (1.54– 3.06)	1.87 (1.60– 2.19)	1.95 (1.67– 2.28)	1.13 (1.07– 1.20)	1.21 (1.15– 1.27)	1.27 (1.18– 1.36)	1.22 (1.13– 1.31)
Q4	1.06 (0.93– 1.20)	1.20 (1.07– 1.36)	1.04 (0.72– 1.49)	1.19 (0.84– 1.70)	1.17 (0.99– 1.39)	1.23 (1.04– 1.46)	1.05 (0.99– 1.11)	1.11 (1.05– 1.17)	1.14 (1.06– 1.22)	1.10 (1.02– 1.18)
Q5 (richest 20%)	ref	ref	ref	ref	ref	ref	ref	ref	ref	ref
**Education**										
presecondary	4.22 (3.87– 4.60)	1.58 (1.44– 1.74)	6.64 (5.23– 8.43)	2.69 (1.98– 3.66)	2.63 (2.36– 2.93)	1.80 (1.59– 2.04)	1.63 (1.57– 1.70)	1.20 (1.14– 1.25)	0.91 (0.86– 0.96)	1.14 (1.07– 1.22)
Secondary	2.14 (1.96– 2.34)	1.40 (1.28– 1.54)	2.72 (2.14– 3.47)	1.90 (1.44– 2.50)	1.60 (1.44– 1.79)	1.44 (1.28– 1.62)	1.36 (1.31– 1.41)	1.19 (1.14– 1.24)	0.94 (0.90– 0.99)	1.04 (1.00– 1.10)
post-secondary	ref	ref	ref	ref	ref	ref	ref	ref	ref	ref
**Country of birth**										
Sweden	ref	ref	ref	ref	ref	ref	ref	ref	ref	ref
Nordic	2.40 (2.19– 2.62)	1.23 (1.14– 1.34)	3.04 (2.40– 3.84)	1.64 (1.32– 2.03)	1.60 (1.41– 1.80)	1.16 (1.04– 1.30)	1.33 (1.27– 1.40)	1.02 (0.98– 1.07)	0.86 (0.80– 0.93)	1.05 (0.97– 1.13)
EU28*	1.65 (1.45– 1.87)	1.14 (1.02– 1.27)	2.21 (1.65– 2.95)	1.47 (1.13– 1.92)	1.18 (1.00– 1.40)	0.98 (0.84– 1.15)	1.16 (1.08– 1.25)	1.02 (0.95– 1.09)	0.95 (0.86– 1.05)	1.04 (0.94– 1.14)
Other	1.58 (1.45– 1.73)	1.52 (1.39– 1.66)	1.91 (1.52– 2.40)	2.03 (1.65– 2.52)	1.15 (1.02– 1.30)	1.22 (1.08– 1.37)	1.10 (1.05– 1.16)	1.02 (0.98– 1.07)	0.98 (0.92– 1.04)	1.09 (1.03– 1.16)

^*1*^PR = Prevalence Ratio; * EU28: includes all EU countries (other than the Nordics) and UKModel 0, unadjusted; Model 1, adjusted for age

Generally, lower income, lower education and being born outside of Sweden, were associated with higher prevalence of problems across all dimensions. For example, the prevalence of problems related to self-care dimension was 6.20 (95% C: 4.45–8.64) times higher for people in the lowest income quintile (vs. highest quintile), 2.69 (95% C: 1.98– 3.66) times higher among people with pre-secondary (vs. post-secondary) education, and 2.03 (95% C: 1.65– 2.52) times higher among those born outside of European union or UK (vs. born in Sweden), adjusted for age (see Table 4 for PR across all dimensions**)**.

### Variations in HRQoL across risk factors and health conditions

**Fig. 4:**
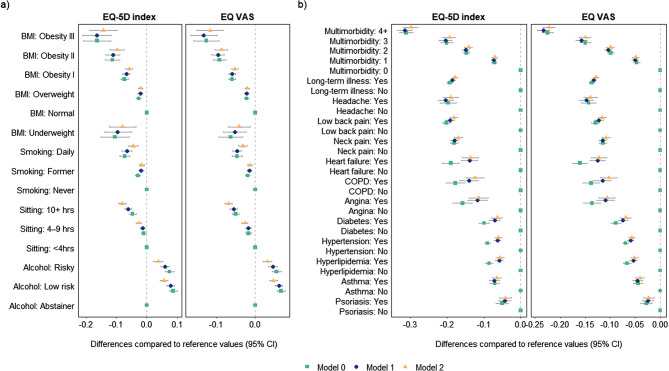
Regression coefficients. (**a**) Relationship between health behavioural risk factors and EQ-5D-3L index and EQ-VAS scores. (**b**) Relationship between health condition and EQ-5D-3L index and EQ-VAS scores. (Model 0, *unadjusted coefficients*; *Model 1*,* adjusted for age; Model 2*,* further adjusted for sex*,* country of birth*,* income and education*)

Generally, people with higher BMI (particularly those classified as having obesity), smokes daily and those with sedentary lifestyle, consistently reported lower HRQoL (on the EQ-5D index as well as the EQ VAS) (Fig. 4a and supplementary Tables **S3** and S4, Additional file 1). Contrarily, risky drinking was associated with higher HRQoL than abstaining. Another aspect worth noting is also that people who reported to have quit smoking daily have EQ-5D index as well as the EQ VAS scores almost at the level of those never smoking daily. The associations remained even after adjusting for sociodemographic differences (see supplementary Table S5, Additional file 1).

Similarly, individuals reporting one or more chronic health conditions had lower HRQoL than those reporting none (Fig. 4b and supplementary Tables S4 & S5, Additional file 1), with multiple conditions linked to even larger reductions in HRQoL. After adjusting for age, sex, country of birth, income, and education, the EQ-5D index was 0.07 points lower (95% CI: − 0.08 to − 0.06) for individuals with one health condition, and 0.30 points lower (95% CI: − 0.32 to − 0.28) for those with 4 or more conditions, compared to those reporting no health conditions. Also, the EQ-5D index of people that reported living with long-term illness was 0.18 points lower (95% CI: -0.19 to -0.17), after adjusting for sociodemographic variables, compared to respondents not reporting long-term illness.

The differences in the mean EQ-5D-3L index values were larger for people who reported different types of chronic pain, heart failure and COPD. For example, the EQ-5D index of people with headaches/migraines was 0.19 points lower (95% CI: -0.21 to -0.17) than those without, after adjusting for differences in sociodemographic profile (Fig. 4b, and supplementary Table S5, Additional file 1).

### Results from supplementary analysis

**Fig. 5:**
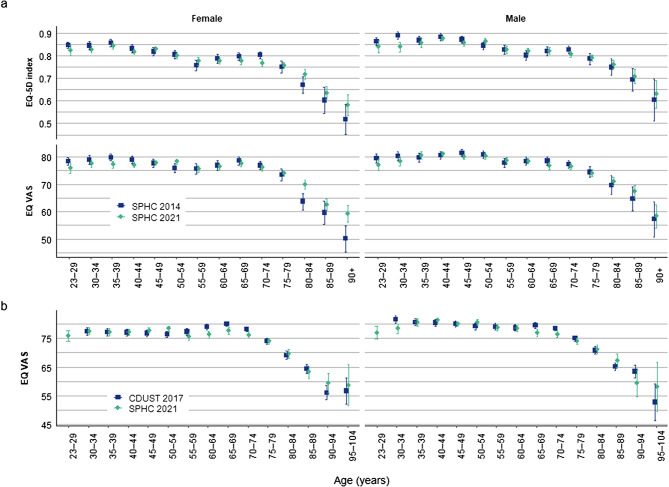
The upper panel (**a)** shows comparison of mean EQ-5D index values and EQ VAS scores, comparing data from 2014 to 2021 in the SPHC. The lower panel (**b**) shows comparisons between EQ VAS scores from SPHC data 2021 (23–104-year-olds) to estimates derived from the CDUST data from 2017 (30–104-year-olds)

Comparisons with alternative data sources were generally reassuring (Fig. 5). Compared with SPHC 2014 data, the EQ-5D index values and EQ VAS scores in our study (SPHC 2021) were largely similar across age groups (Fig. 5a). Age stratified comparisons of prevalences of reported problems across EQ-5D dimensions are also presented in the supplementary (supplementary Tables S7, Additional file 1). Self-rated EQ VAS scores from our data also aligned to estimates using 2017 data from five central Swedish regions, except for slightly higher EQ VAS scores among females aged 60–70 and males aged 30–34 and 65–74 (Fig. 5b).

Comparing EQ-5D index values from the two value sets showed that the Swedish experience-based set produced higher estimates in every age group, as anticipated. A figure illustrating this gap and a supplementary table offers age- and sex-specific EQ-5D index estimates based on the experience-based value set for practitioners and analysts interested in norms derived from the experience-based value set (see supplementary figure S1 and Table Type="Bold">S8, Additional file 1). Similarly, our findings were also robust when a two-part regression model was used instead of the linear model was (see supplementary Table S6, Additional file 1).

## Discussion

This study described the HRQoL of the adult general population in Stockholm County, and explored variations in HRQoL across different sociodemographic groups, and groups with behavioural and metabolic risk factors, as well as chronic health conditions. The estimates of the mean EQ-5D index, EQ VAS, and prevalence of health problems across the different EQ-5D dimensions could serve as valuable reference data for identifying health gaps, evaluating and monitoring policy changes, assessing the value of therapeutic and public health interventions, and informing future investment decisions.

### Summary of main findings

Overall, a high proportion of the adult general population reported good health. While this is in line with previous studies, the EQ-5D is known to have high ceiling effects and minor health problems might not have been captured in the estimates of prevalence of health problems across dimensions or in the mean EQ-5D index values [46]. Generally, pain/discomfort and anxiety/depression were the most predominant issues in the adult general population of Stockholm County. Also, as anticipated and in line with earlier findings, the prevalence of problems across all dimensions was generally higher in older age groups – though the pattern of reported problems varied substantially over the life course. The anxiety/depression dimension constitutes an exception to this trend. Its prevalence was highest among younger individuals, though it rose again among the oldest old.

The mean EQ-5D index values generally declined across age groups; however, this decline is less evident among younger adults. Notably, values are slightly higher in those aged 35–44 compared to 23–34-year-olds, then remain relatively stable until around age 55, after which a clear decline is observed, most pronounced among individuals aged 85 and above. The lower values observed among the younger age groups are likely attributable to the higher proportions reporting problems in the anxiety/depression dimension.

The results also align with previous studies, showing that HRQoL varies substantially across sociodemographic conditions, risk factors and by chronic health conditions [15, 16, 23, 34, 42, 43, 47–49]. Age adjusted EQ-5D index values and EQ VAS scores were lower for people with lower education and for those in the lower income quintiles. Further, while the magnitude was small, the age adjusted mean EQ-5D index values and EQ VAS scores tended to be lower among people born outside Sweden, particularly those born outside of the EU28 and Nordics (as categorised ‘*other’*).

Persistent associations between behavioural risk factors and HRQoL were also observed. Having higher BMI, smoking daily, sitting longer hours per day was linked to substantially lower HRQoL. While no causal conclusion can be drawn, the findings are in line with previous research [42, 43, 47, 48, 50]. Alcohol consumption on the other hand was associated with higher HRQoL. While this finding is also in line with previous studies [42, 47], this may result from individuals being classified as abstainers when, in fact, they refrain from alcohol because of underlying health problems. Additionally, as anticipated and similar to previous studies [21, 23, 24, 49], having any chronic health condition was associated with lower HRQoL. Particularly, those with long-term illness or multiple health conditions exhibited poorer HRQoL.

### Limitations

This study used a substantially larger sample size than most prior research and utilized calibration with auxiliary variables from registry data to generate weights that enhance representativeness of the population of Stockholm County. However, as with all studies, some limitations warrant consideration. One potential limitation is that, because data collection took place during the COVID-19 pandemic, the results may overestimate the prevalence of some self-reported problems, either as a consequence of COVID-19 infection itself or due to the broader circumstances surrounding the pandemic [51, 52]. There is also the possibility of differential pandemic effects, whereby older individuals and high-risk groups were more likely to experience severe COVID-19, which may have influenced their participation and reported HRQoL. Moreover, individuals with lower socioeconomic status were disproportionately affected (for example, due to more limited opportunities to work from home), which may have contributed to an overestimation of the social disparities observed in our study. Moreover, although the sample size was large and calibration weights were applied, there still is a potential residual selection bias. This could have led to individuals who are generally less likely to respond to surveys may be further underrepresented, and continued participation in the cohort may reflect a healthier subset of the population, partly due to mortality selection or non-participation among those who were severely ill. Nonetheless, comparing the estimates in this study with two large population-based pre-pandemic data sources did not reveal major deviations.

Additionally, while sociodemographic variables were retrieved from national registers, other exposures such as the risk factors and health conditions were self-reported, which might have introduced some measurement errors. Likewise, this study relied on a predetermined list of conditions available in the survey, which may have excluded some clinically relevant diagnoses. Moreover, categorising health conditions as binary is crude and may not adequately capture all meaningful variations in HRQoL across severity and duration of illness.

Finally, modelling EQ-5D index and EQ VAS using linear models is often considered as suboptimal approach given the non-standard response distributions of the index values and EQ VAS scores. Nonetheless, a sensitivity analysis using a two-part regression model showed similar patterns in the EQ-5D index (see supplementary Table S6 in the Additional file 1). and, previous studies comparing OLS, against alternative estimators for utility estimation found no single model demonstrating clear superiority [47, 53].

### Implications for policy and future research

This study has several implications for clinical and population health assessments, funding priorities, and future research. First, the results may serve as a benchmark for comparing patient-reported HRQoL data with those of the general population to identify the burden of disease and care needs of different patient groups. This is particularly relevant, as the regions in Sweden are increasingly seeking methods to estimate and compare population healthcare needs to better align resource allocation decisions with those needs. The results also provide valuable inputs for future model-based cost-utility analyses, to follow-up public health programmes and guiding future investments. Given the variations in EQ-5D index, EQ VAS and the levels of severity across individual and population characteristics, comparisons should be made to population groups of similar (age and sex) characteristics. The age and sex specific estimates in Table 3 and supplementary Tables S1 & S2 in the Additional file 1, can be used for such applications. Efforts have been made to make the results generalisable to the adult population in Stockholm County, but they could also be applicable on the national level, and to other countries with comparable sociodemographic context (*see* Table 1).

Second, EQ-5D is routinely collected in several of Swedish national quality registries (NQR). Although these data could provide a valuable basis for estimating severity of illness across different patient groups and therapeutic areas [25, 54], which in turn could be valuable priority setting and healthcare planning and evaluation for healthcare providers, only 50% of the registries have reported that the EQ-5D data they collect is being used for any kind of follow-up or decision-making [25]. Findings of this study can be used as reference values facilitating interpretation of patient outcomes.

Third, the results could, in combination with mortality estimates, provide valuable quantitative support for estimations of disease severity, an important component in adjusting for the need of patients in HTA and priority-setting decisions as well as developing clinical guidelines [19, 55].

Fourth, the study has provided evidence, supporting existing knowledge, of a consistent association between individuals’ socioeconomic position and HRQoL. Such findings are interesting per se, but they also strengthen the evidence regarding the validity of the EQ-5D-3L for use in studying health inequalities. The fact that people living with a chronic health conditions or multiple morbidity reported substantially lower HRQoL also underscore the need to consider general HRQoL in addition to clinical symptoms during clinical follow-ups.

Some ongoing methodological discussions should be considered in future use of the results as population norms, and in interpreting socioeconomic differentials and variations across health risk groups. First, there are discussions about what is a minimally important differences (MID) I– the smallest amount of difference that patients can recognize and value – in different measures of HRQoL. MIDs vary greatly depending on compositional and contextual factors, and the methodologies used to determine them [56–58]. Whether MIDs should be used to interpret values from preference weighted instruments is also an area of active debate. Similarly, considerations should also be taken to the value set used to calculate the EQ-5D index [15, 35, 38, 59], in interpreting and using the estimates or making comparisons. For e.g., compared to the UK value set used in this study, the experience-based Swedish value set is known to yield higher estimates of health-related quality of life [35] (see also supplementary Figure S1 and Table S8**,** in the additional file 1). Similarly, the wording of the EQ-5D-3L, particularly for the anxiety/depression dimension, differs slightly and does not imply a clinical diagnosis of anxiety or depression, but rather reflects self-reported problems related to worry and low mood.

Developing quality adjusted life expectancy norms was considered beyond the scope for this study. However, future studies could link the SPHC with mortality data to allow for a quantification of quality-adjusted life expectancy of the general population. Future studies could also look more into temporal trends in health inequalities in HRQoL, exploring if there are widening gaps similar to those found for life-expectancy and all-cause mortality [11, 12].

## Conclusion

This study describes the health-related quality of life (HRQoL) of the adult general population in Stockholm County. While HRQoL was generally good, it varied across sociodemographic groups. Particularly, female respondents, older adults, and individuals with lower socioeconomic status reported lower HRQoL. Substantial variations were also observed across behavioural risk factors and health conditions. These results should be interpreted as characterizing differences across risk groups rather than indicating causal relationships, but they can nonetheless inform the identification of health gaps and support the evaluation and monitoring of policy interventions. EQ-5D-3L population norms can serve as a reference to support reimbursement decisions and guide healthcare priority setting.

## Supplementary Information

Below is the link to the electronic supplementary material.

Supplementary Material 1

## Data Availability

The data that support the findings of this study cannot be shared according to the Swedish law and GDPR regulations. Read more on the SPHC website for information on how to request for access to the SPHC data: Hälsa Stockholm - för forskare (https://www.ces.regionstockholm.se/projekt-och-uppdrag/halsa-stockholm/SPHC-data/).

## Abbreviations

AD: Anxiety/Depression
AUDIT-C: Alcohol Use Disorders Identification Test – Consumption
BMI: Body Mass Index
COPD: Chronic Obstructive Pulmonary Disease
COVID-19: Coronavirus Disease 2019
EQ VAS: EuroQol Visual Analogue Scale
EU28: Individuals born in the EU27 countries or the United Kingdom
HRQoL: Health-Related Quality of Life
HTA: Health Technology Assessment
MCID: Minimally Clinically Important Differences
MO: Mobility
NQR: National Quality Registries
OLS regression: Ordinary Least Squares Regression
PD: Pain/Discomfort
PR: Prevalence Ratios
PROM: Patient-Reported Outcome Measure
QALYs: Quality-Adjusted Life Years
SC: Self-Care
SPHC: Stockholm Public Health Cohort
UA: Usual Activities
UK: United Kingdom
