# Supplementary material for: Variations in health-related quality of life across sociodemographic groups, health conditions, and modifiable risk factors: a population-based EQ-5D-3L study
Source: Health Qual Life Outcomes. 2026 Jul 20;24:96. doi: 10.1186/s12955-026-02587-9 (PMC13386571; doi:10.1186/s12955-026-02587-9)
Supplement: Supplementary file 1 — Supplementary Material 1 [file 12955_2026_2587_MOESM1_ESM.docx]

**Additional file 1**

**Table S1**: Distribution of responses (Proportion (95% CI)) to EQ-5D-3L dimensions across different sociodemographic charcterstics.

|  |  | **Mobility** | **Self-care** | **Usua activity** | **Pain/discomfort** | **Anxiety/depression,** |
| --- | --- | --- | --- | --- | --- | --- |
| **Sex** | Male, n=22225 | 13.68 (13–14) | 2.52 (2.2–2.9) | 9.77 (9.1–11) | 43.04 (42–44) | 34.93 (34–36) |
|  | Female, n=30068 | 16.40 (16–17) | 3.06 (2.7–3.4) | 13.64 (13–14) | 51.62 (51–53) | 43.05 (42–44) |
| **Age** | 23–29, n=680 | 3.16 (2.0–5.0) | 1.03 (0.43–2.4) | 10.11 (7.8–13) | 29.59 (26–34) | 55.60 (51–60) |
|  | 30–34, n=1234 | 4.79 (3.5–6.5) | 0.80 (0.34–1.8) | 9.04 (7.4–11) | 34.67 (32–38) | 49.30 (46–52) |
|  | 35–39, n=1839 | 4.93 (3.7–6.6) | 0.75 (0.39–1.4) | 7.29 (5.9–9.0) | 33.54 (31–37) | 43.72 (41–47) |
|  | 40–44, n=3033 | 5.29 (4.3–6.4) | 0.84 (0.51–1.4) | 7.52 (6.5–8.7) | 38.77 (37–41) | 39.21 (37–41) |
|  | 45–49, n=4055 | 7.51 (6.4–8.8) | 1.24 (0.85–1.8) | 7.90 (6.9–9.0) | 41.66 (40–44) | 36.45 (35–38) |
|  | 50–54, n=4956 | 10.37 (9.0–12) | 2.11 (1.5–3.0) | 8.67 (7.5–10) | 47.81 (46–50) | 33.83 (32–36) |
|  | 55–59, n=5563 | 15.25 (14–17) | 3.55 (2.7–4.7) | 11.70 (10–13) | 52.87 (51–55) | 35.79 (34–38) |
|  | 60–64, n=5500 | 18.91 (17–21) | 2.79 (2.0–3.8) | 12.81 (11–14) | 57.29 (55–59) | 33.30 (31–35) |
|  | 65–69, n=5652 | 22.06 (20–24) | 2.96 (2.2–4.0) | 12.06 (11–14) | 57.58 (55–60) | 31.35 (29–34) |
|  | 70–74, n=6457 | 26.16 (24–28) | 4.08 (3.2–5.1) | 12.81 (11–14) | 61.81 (60–64) | 30.54 (29–33) |
|  | 75–79, n=6773 | 32.02 (30–34) | 5.34 (4.3–6.5) | 17.30 (16–19) | 64.58 (63–67) | 30.37 (29–32) |
|  | 80–84, n=4006 | 43.84 (41–47) | 7.37 (5.9–9.2) | 23.14 (21–26) | 69.27 (67–72) | 32.86 (30–35) |
|  | 85–89, n=1699 | 60.86 (57–65) | 12.64 (10–16) | 37.27 (33–41) | 77.36 (74–80) | 41.14 (37–45) |
|  | 90+, n=846 | 74.62 (70–79) | 29.31 (25–34) | 47.48 (42–53) | 79.62 (75–84) | 39.65 (35–45) |
| **Income** | Q1, n=5269 | 26.06 (24–28) | 7.17 (6.1–8.4) | 23.63 (22–26) | 55.77 (53–58) | 50.70 (48–53) |
|  | Q2, n=10643 | 20.97 (20–22) | 4.18 (3.6–4.8) | 15.52 (14–17) | 52.85 (51–55) | 43.96 (42–46) |
|  | Q3, n=11013 | 12.95 (12–14) | 1.78 (1.4–2.2) | 10.26 (9.3–11) | 46.31 (45–48) | 38.21 (37–40) |
|  | Q4, n=12315 | 9.06 (8.3–9.9) | 0.97 (0.76–1.2) | 6.41 (5.7–7.2) | 42.76 (41–44) | 34.26 (33–36) |
|  | Q5, n=13053 | 8.55 (7.8–9.3) | 0.93 (0.72–1.2) | 5.47 (4.8–6.2) | 40.84 (39–42) | 30.14 (29–32) |
| **Education** | L1, n=4885 | 32.61 (31–34) | 7.22 (6.3–8.2) | 20.76 (19–22) | 62.86 (61–65) | 36.69 (35–38) |
|  | L2, n=18698 | 16.55 (16–17) | 2.96 (2.6–3.4) | 12.65 (12–14) | 52.32 (51–54) | 38.29 (37–40) |
|  | L3, n=28710 | 7.73 (7.2–8.3) | 1.09 (0.89–1.3) | 7.89 (7.3–8.6) | 38.53 (37–40) | 40.53 (39–42) |
| **Country of birth** | Sweden, n=45128 | 12.32 (12–13) | 2.07 (1.8–2.3) | 10.95 (10–12) | 45.32 (44–46) | 39.68 (39–41) |
|  | Nordic, n=2473 | 29.52 (27–32) | 6.29 (5.1–7.7) | 17.48 (16–20) | 60.42 (58–63) | 34.18 (32–37) |
|  | EU28, n=1621 | 20.32 (18–23) | 4.57 (3.5–5.9) | 12.95 (11–15) | 52.62 (49–56) | 37.73 (34–41) |
|  | other, n=3071 | 19.48 (18–21) | 3.96 (3.3–4.8) | 12.65 (11–14) | 50.06 (48–52) | 38.80 (37–41) |

**Table S2.** Proportion and 95 % CI by EQ-5D-3L dimension, level of impairment, age groups and Sex.

|  |  | **Overall** | **23–34** | **35–44** | **45–54** | **55–64** | **65–84** | **85+** |
| --- | --- | --- | --- | --- | --- | --- | --- | --- |
| ***All*** | **Mobility**, % |  |  |  |  |  |  |  |
|  | No problem | 85.0 (85–86) | 95.9 (95–97) | 94.9 (94–96) | 91.5 (90–92) | 82.9 (82–84) | 70.3 (69–71) | 33.9 (31–37) |
|  | Moderate | 14.7 (14–15) | 4.0 (3.1–5.2) | 4.9 (4.2–5.8) | 8.5 (7.6–9.5) | 17.0 (16–18) | 29.3 (28–30) | 64.7 (62–68) |
|  | Severe | 0.2 (0.15–0.30) | 0.1 (0.01–0.74) | 0.2 (0.07–0.44) | 0.1 (0.03–0.15) | 0.1 (0.04–0.26) | 0.4 (0.24–0.59) | 1.4 (0.83–2.3) |
|  | **Self-care**, % |  |  |  |  |  |  |  |
|  | No problem | 97.3 (97–98) | 99.2 (99–100) | 99.2 (99–99) | 98.5 (98–99) | 97.0 (96–98) | 95.4 (95–96) | 81.8 (79–84) |
|  | Moderate | 2.2 (2.0–2.4) | 0.6 (0.29–1.1) | 0.7 (0.48–1.1) | 1.3 (0.96–1.7) | 2.6 (2.1–3.3) | 3.7 (3.3–4.3) | 14.7 (12–17) |
|  | Severe | 0.5 (0.38–0.60) | 0.2 (0.05–0.86) | 0.1 (0.02–0.34) | 0.2 (0.14–0.39) | 0.4 (0.23–0.73) | 0.9 (0.65–1.2) | 3.5 (2.4–4.9) |
|  | **Usual activities**, % |  |  |  |  |  |  |  |
|  | No problem | 88.4 (88–89) | 90.6 (89–92) | 92.8 (92–94) | 91.8 (91–93) | 87.7 (87–89) | 84.6 (84–85) | 59.1 (56–62) |
|  | Moderate | 10.3 (9.8–11) | 8.3 (6.9–9.8) | 6.4 (5.6–7.4) | 7.1 (6.3–7.9) | 11.1 (10–12) | 14.0 (13–15) | 33.8 (31–37) |
|  | Severe | 1.3 (1.2–1.5) | 1.2 (0.71–1.9) | 0.8 (0.56–1.1) | 1.1 (0.85–1.4) | 1.2 (0.91–1.6) | 1.4 (1.1–1.7) | 7.1 (5.6–9.0) |
|  | **Pain/discomfort**, % |  |  |  |  |  |  |  |
|  | No problem | 52.7 (52–54) | 67.4 (65–70) | 63.6 (62–65) | 56.2 (55–58) | 44.9 (43–46) | 37.3 (36–38) | 21.8 (19–25) |
|  | Moderate | 44.3 (43–45) | 31.2 (29–34) | 35.3 (34–37) | 41.8 (40–43) | 50.7 (49–52) | 57.9 (57–59) | 68.8 (66–72) |
|  | Severe | 3.0 (2.7–3.2) | 1.4 (0.91–2.2) | 1.1 (0.81–1.5) | 2.0 (1.6–2.6) | 4.4 (3.7–5.1) | 4.8 (4.3–5.4) | 9.5 (7.7–12) |
|  | **Anxiety/depression**, % |  |  |  |  |  |  |  |
|  | No problem | 60.9 (60–62) | 48.1 (45–51) | 59.0 (57–61) | 64.5 (63–66) | 65.6 (64–67) | 68.8 (68–70) | 59.8 (57–63) |
|  | Moderate | 35.6 (35–36) | 46.0 (43–49) | 37.2 (35–39) | 32.6 (31–34) | 31.1 (30–33) | 29.6 (29–31) | 36.6 (33–40) |
|  | Severe | 3.5 (3.2–3.9) | 6.0 (4.8–7.3) | 3.8 (3.1–4.7) | 2.9 (2.4–3.4) | 3.3 (2.7–4.0) | 1.6 (1.3–1.9) | 3.5 (2.4–5.2) |
| ***Female*** | **Mobility**, % |  |  |  |  |  |  |  |
|  | No problem | 83.8 (83–84) | 96.8 (96–98) | 93.7 (92–95) | 90.8 (89–92) | 80.9 (79–83) | 69.1 (68–71) | 30.6 (27–35) |
|  | Moderate | 16.0 (15–17) | 3.2 (2.3–4.5) | 6.1 (5.0–7.4) | 9.1 (8.0–10) | 19.0 (17–21) | 30.5 (29–32) | 68.0 (64–72) |
|  | Severe | 0.2 (0.14–0.31) | 0.0 (0.00–0.00) | 0.2 (0.04–0.64) | 0.1 (0.04–0.28) | 0.1 (0.03–0.45) | 0.4 (0.22–0.72) | 1.4 (0.66–2.8) |
|  | **Self-care**, % |  |  |  |  |  |  |  |
|  | No problem | 97.0 (97–97) | 99.6 (99–100) | 98.7 (98–99) | 98.1 (97–99) | 96.4 (95–97) | 95.6 (95–96) | 80.0 (76–83) |
|  | Moderate | 2.5 (2.2–2.8) | 0.4 (0.15–1.1) | 1.1 (0.71–1.8) | 1.6 (1.1–2.3) | 3.3 (2.4–4.4) | 3.4 (2.8–4.0) | 16.4 (13–20) |
|  | Severe | 0.5 (0.40–0.65) | 0.0 (0.00–0.00) | 0.2 (0.03–0.67) | 0.3 (0.14–0.49) | 0.3 (0.15–0.66) | 1.1 (0.75–1.6) | 3.7 (2.3–5.8) |
|  | **Usual activities**, % |  |  |  |  |  |  |  |
|  | No problem | 86.4 (86–87) | 89.4 (87–91) | 90.0 (88–91) | 89.9 (89–91) | 85.5 (84–87) | 84.2 (83–85) | 54.3 (50–58) |
|  | Moderate | 11.9 (11–13) | 9.3 (7.5–11) | 8.7 (7.4–10) | 8.8 (7.7–10) | 12.9 (11–14) | 14.4 (13–16) | 38.0 (34–42) |
|  | Severe | 1.6 (1.4–1.9) | 1.3 (0.73–2.4) | 1.4 (0.94–1.9) | 1.3 (0.97–1.7) | 1.6 (1.1–2.2) | 1.4 (1.1–1.9) | 7.7 (5.7–10) |
|  | **Pain/discomfort**, % |  |  |  |  |  |  |  |
|  | No problem | 48.5 (47–50) | 64.4 (61–68) | 58.8 (56–61) | 51.6 (50–54) | 40.6 (39–43) | 33.9 (32–35) | 16.5 (14–20) |
|  | Moderate | 47.6 (47–49) | 34.1 (31–37) | 39.6 (37–42) | 45.6 (44–48) | 53.6 (52–56) | 60.0 (58–62) | 72.5 (69–76) |
|  | Severe | 3.9 (3.5–4.3) | 1.5 (0.82–2.6) | 1.6 (1.1–2.2) | 2.8 (2.2–3.6) | 5.8 (4.8–7.0) | 6.1 (5.3–7.0) | 11.0 (8.6–14) |
|  | **Anxiety/depression**, % |  |  |  |  |  |  |  |
|  | No problem | 57.0 (56–58) | 45.5 (42–49) | 53.5 (51–56) | 60.3 (58–62) | 62.1 (60–64) | 64.8 (63–66) | 55.8 (52–60) |
|  | Moderate | 39.3 (38–40) | 48.8 (45–52) | 42.6 (40–45) | 36.3 (34–38) | 34.0 (32–36) | 33.5 (32–35) | 40.5 (36–45) |
|  | Severe | 3.7 (3.3–4.2) | 5.7 (4.4–7.4) | 3.9 (3.1–4.9) | 3.4 (2.7–4.2) | 3.9 (3.1–4.9) | 1.7 (1.3–2.2) | 3.6 (2.3–5.8) |
| ***Male*** | **Mobility**, % |  |  |  |  |  |  |  |
|  | No problem | 86.4 (86–87) | 94.9 (93–96) | 96.1 (95–97) | 92.1 (91–93) | 84.9 (83–87) | 71.5 (70–73) | 39.9 (35–45) |
|  | Moderate | 13.4 (13–14) | 4.9 (3.4–7.0) | 3.7 (2.7–5.1) | 7.9 (6.5–9.4) | 15.0 (13–17) | 28.1 (27–30) | 58.7 (54–64) |
|  | Severe | 0.2 (0.12–0.38) | 0.2 (0.03–1.6) | 0.2 (0.06–0.65) | 0.0 (0.01–0.13) | 0.1 (0.03–0.29) | 0.4 (0.18–0.68) | 1.4 (0.73–2.8) |
|  | **Self-care**, % |  |  |  |  |  |  |  |
|  | No problem | 97.6 (97–98) | 98.8 (97–99) | 99.7 (99–100) | 98.8 (98–99) | 97.6 (97–98) | 95.2 (94–96) | 85.1 (81–88) |
|  | Moderate | 1.9 (1.7–2.3) | 0.8 (0.30–1.9) | 0.3 (0.14–0.80) | 1.0 (0.59–1.7) | 1.9 (1.3–2.8) | 4.2 (3.5–5.0) | 11.8 (8.9–15) |
|  | Severe | 0.4 (0.29–0.66) | 0.5 (0.11–1.8) | 0.0 (0.00–0.00) | 0.2 (0.08–0.48) | 0.5 (0.22–1.1) | 0.6 (0.38–1.1) | 3.1 (1.9–4.9) |
|  | **Usual activities**, % |  |  |  |  |  |  |  |
|  | No problem | 90.4 (90–91) | 91.9 (89–94) | 95.6 (94–97) | 93.7 (92–95) | 89.8 (88–91) | 85.0 (84–86) | 67.7 (63–72) |
|  | Moderate | 8.6 (7.9–9.3) | 7.2 (5.3–9.6) | 4.2 (3.3–5.4) | 5.4 (4.4–6.6) | 9.3 (8.0–11) | 13.6 (12–15) | 26.3 (22–31) |
|  | Severe | 1.0 (0.80–1.3) | 1.0 (0.42–2.2) | 0.2 (0.08–0.61) | 0.9 (0.57–1.5) | 0.9 (0.53–1.5) | 1.3 (0.97–1.8) | 6.0 (4.1–8.8) |
|  | **Pain/discomfort**, % |  |  |  |  |  |  |  |
|  | No problem | 57.2 (56–58) | 70.7 (67–74) | 68.3 (66–71) | 60.6 (58–63) | 49.2 (47–51) | 41.1 (39–43) | 31.2 (27–36) |
|  | Moderate | 40.7 (40–42) | 27.9 (24–32) | 31.1 (28–34) | 38.1 (36–40) | 47.8 (46–50) | 55.5 (54–57) | 62.1 (57–67) |
|  | Severe | 2.1 (1.8–2.4) | 1.4 (0.70–2.7) | 0.6 (0.32–1.2) | 1.3 (0.83–2.1) | 2.9 (2.2–3.9) | 3.3 (2.8–4.0) | 6.7 (4.6–9.8) |
|  | **Anxiety/depression**, % |  |  |  |  |  |  |  |
|  | No problem | 65.0 (64–66) | 50.9 (47–55) | 64.5 (62–67) | 68.5 (66–71) | 69.2 (67–71) | 73.3 (72–75) | 67.1 (62–72) |
|  | Moderate | 31.6 (30–33) | 42.8 (39–47) | 31.7 (29–34) | 29.1 (27–31) | 28.2 (26–30) | 25.3 (24–27) | 29.6 (25–34) |
|  | Severe | 3.3 (2.8–3.9) | 6.3 (4.5–8.6) | 3.8 (2.6–5.4) | 2.4 (1.8–3.2) | 2.6 (1.8–3.7) | 1.4 (1.0–1.9) | 3.3 (1.6–6.8) |

**Table S3:** Prevalence and 95% confidence interval of any level of impairment by EQ-5D-3L dimensions across risk factors and health conditions

|  |  | **Mobility** | **Self-care** | **Usua activity** | **Pain/discomfort** | **Anxiety/depression** |
| --- | --- | --- | --- | --- | --- | --- |
| BMI group | Underweight, n=705 | 16.80 (12–22) | 6.25 (3.4–11) | 20.51 (15–28) | 48.97 (41–57) | 57.61 (50–65) |
|  | Normal, n=24894 | 9.52 (8.9–10) | 1.82 (1.6–2.1) | 9.20 (8.5–9.9) | 40.42 (39–42) | 38.48 (37–40) |
|  | Overweight, n=18592 | 16.14 (15–17) | 2.61 (2.2–3.0) | 11.65 (11–13) | 50.66 (49–52) | 36.83 (36–38) |
|  | Obesity class 1, n=5543 | 27.01 (25–29) | 5.12 (4.1–6.3) | 16.50 (15–18) | 59.78 (57–62) | 42.75 (40–45) |
|  | Obesity class 2, n=1296 | 31.53 (28–36) | 7.13 (4.8–10) | 22.23 (18–26) | 65.91 (61–70) | 46.55 (42–51) |
|  | Obesity class 3, n=472 | 43.07 (36–51) | 6.85 (4.3–11) | 28.32 (22–35) | 70.50 (62–78) | 47.99 (40–56) |
| Smoking | Daily, n=2727 | 24.41 (22–27) | 4.51 (3.5–5.8) | 18.02 (16–21) | 58.25 (55–61) | 47.28 (44–50) |
|  | Quit smoking, n=15221 | 19.04 (18–20) | 3.04 (2.6–3.5) | 13.79 (13–15) | 55.43 (54–57) | 38.33 (37–40) |
|  | Never, n=33929 | 12.86 (12–13) | 2.53 (2.2–2.8) | 10.54 (10–11) | 43.91 (43–45) | 38.68 (38–40) |
| Alcohol consumption | Abstained, n=5832 | 25.85 (24–28) | 7.65 (6.6–8.8) | 20.99 (19–23) | 55.61 (53–58) | 43.88 (42–46) |
|  | Not hazardous, n=38035 | 13.28 (13–14) | 1.86 (1.7–2.1) | 9.96 (9.4–11) | 45.95 (45–47) | 36.68 (36–38) |
|  | Hazardous, n=7988 | 11.29 (10–12) | 1.69 (1.3–2.2) | 9.88 (8.7–11) | 45.22 (43–47) | 44.69 (42–47) |
| Sitting (hours/day) | <4, n=7674 | 13.84 (13–15) | 2.39 (1.9–3.0) | 8.95 (7.9–10) | 47.86 (46–50) | 32.11 (30–34) |
|  | 04–09, n=34140 | 14.85 (14–15) | 2.22 (2.0–2.5) | 10.98 (10–12) | 47.97 (47–49) | 36.71 (36–38) |
|  | 10+, n=10040 | 16.06 (15–17) | 4.27 (3.7–4.9) | 15.28 (14–17) | 45.77 (44–48) | 49.07 (47–51) |
| **Self-reported health conditions** | |  |  |  |  |  |
| Neck pain | No, n=43316 | 12.37 (12–13) | 1.97 (1.8–2.2) | 8.77 (8.3–9.3) | 40.49 (40–41) | 34.61 (34–36) |
|  | Yes, n=8572 | 26.91 (25–29) | 6.33 (5.5–7.3) | 25.35 (24–27) | 79.98 (78–82) | 60.51 (59–62) |
| Low back pain | No, n=42721 | 10.47 (10–11) | 1.80 (1.6–2.0) | 8.29 (7.8–8.8) | 40.05 (39–41) | 35.74 (35–37) |
|  | Yes, n=9165 | 38.33 (37–40) | 7.72 (6.8–8.8) | 29.21 (28–31) | 85.87 (84–87) | 56.79 (55–59) |
| Headache/migraine | No, n=49383 | 13.97 (13–14) | 2.45 (2.2–2.7) | 10.31 (9.8–11) | 45.41 (45–46) | 36.90 (36–38) |
|  | Yes, n=2675 | 29.28 (26–32) | 7.06 (5.6–8.9) | 31.53 (28–35) | 75.62 (72–79) | 71.08 (68–74) |
| **Physician diagnosed conditions** | |  |  |  |  |  |
| Diabetes | No, n=47016 | 13.16 (13–14) | 2.33 (2.1–2.6) | 10.64 (10–11) | 45.77 (45–47) | 38.94 (38–40) |
|  | Yes, n=4456 | 37.61 (35–40) | 8.21 (6.9–9.8) | 24.68 (23–27) | 65.47 (63–68) | 39.71 (37–42) |
| Angina pectoris | No, n=49041 | 13.62 (13–14) | 2.41 (2.2–2.7) | 10.86 (10–11) | 46.19 (45–47) | 38.87 (38–40) |
|  | Yes, n=1903 | 49.10 (45–53) | 12.24 (9.9–15) | 32.12 (29–36) | 73.98 (70–77) | 45.41 (42–49) |
| Heart failure | No, n=48622 | 13.24 (13–14) | 2.28 (2.1–2.5) | 10.49 (10–11) | 46.00 (45–47) | 38.69 (38–40) |
|  | Yes, n=2427 | 55.04 (52–59) | 14.19 (12–17) | 39.42 (36–43) | 77.66 (74–81) | 49.13 (46–53) |
| Hypertention | No, n=34089 | 9.43 (8.9–10) | 1.78 (1.5–2.0) | 9.18 (8.6–9.8) | 41.66 (41–43) | 39.59 (39–41) |
|  | Yes, n=17990 | 33.79 (33–35) | 6.07 (5.5–6.7) | 20.16 (19–21) | 66.85 (66–68) | 37.52 (36–39) |
| Asthma | No, n=46461 | 14.07 (14–15) | 2.49 (2.3–2.7) | 10.85 (10–11) | 45.62 (45–46) | 38.21 (37–39) |
|  | Yes, n=4713 | 20.87 (19–23) | 4.89 (3.9–6.0) | 17.98 (16–20) | 60.98 (58–64) | 47.04 (44–50) |
| COPD | No, n=49457 | 13.81 (13–14) | 2.44 (2.2–2.7) | 10.90 (10–11) | 46.31 (45–47) | 38.74 (38–40) |
|  | Yes, n=1598 | 51.87 (48–56) | 13.70 (11–17) | 36.67 (33–41) | 77.68 (74–81) | 49.94 (46–54) |
| Psoriasis | No, n=48717 | 14.32 (14–15) | 2.57 (2.3–2.8) | 11.25 (11–12) | 46.50 (46–47) | 38.92 (38–40) |
|  | Yes, n=2249 | 22.15 (19–25) | 5.70 (4.1–7.9) | 17.39 (15–21) | 61.23 (57–65) | 41.97 (38–46) |
| Hyperlipidemia | No, n=42848 | 12.46 (12–13) | 2.20 (2.0–2.5) | 10.43 (9.9–11) | 44.43 (44–45) | 38.93 (38–40) |
|  | Yes, n=8306 | 32.64 (31–34) | 6.58 (5.6–7.7) | 19.89 (18–22) | 68.34 (67–70) | 39.52 (38–41) |
| **Long-term illness** | No, n=31431 | 5.93 (5.5–6.4) | 0.59 (0.45–0.76) | 3.18 (2.8–3.6) | 33.21 (32–34) | 31.95 (31–33) |
|  | Yes, n=20501 | 32.37 (31–34) | 7.02 (6.4–7.7) | 28.22 (27–29) | 74.55 (73–76) | 52.75 (51–54) |
| **Self-rated health** | Very good, n=11438 | 1.56 (1.3–1.9) | 0.23 (0.09–0.61) | 0.74 (0.49–1.1) | 16.38 (15–18) | 14.64 (13–16) |
|  | Good, n=25761 | 8.01 (7.5–8.6) | 0.75 (0.61–0.93) | 4.35 (3.9–4.9) | 43.79 (43–45) | 35.16 (34–36) |
|  | Neither, n=12350 | 34.09 (33–36) | 5.07 (4.4–5.8) | 26.57 (25–28) | 78.90 (77–80) | 63.72 (62–65) |
|  | Bad, n=2243 | 62.21 (58–66) | 20.63 (18–23) | 68.72 (65–72) | 91.55 (89–94) | 84.66 (82–87) |
|  | Very bad, n=338 | 79.01 (68–87) | 45.94 (36–56) | 87.52 (79–93) | 94.06 (86–98) | 91.79 (85–96) |
| **Multimorbidity (number of conditions)** | 0, n=20379 | 5.00 (4.5–5.5) | 0.84 (0.63–1.1) | 4.70 (4.2–5.3) | 27.92 (27–29) | 32.96 (32–34) |
|  | 1, n=14322 | 13.11 (12–14) | 1.91 (1.6–2.3) | 10.58 (9.6–12) | 53.67 (52–55) | 39.52 (38–41) |
|  | 2, n=9008 | 24.96 (23–27) | 3.83 (3.2–4.6) | 18.43 (17–20) | 70.15 (68–72) | 46.42 (45–48) |
|  | 3, n=4883 | 38.06 (36–41) | 7.80 (6.4–9.5) | 27.18 (25–30) | 80.09 (78–82) | 48.44 (46–51) |
|  | 4+, n=3677 | 56.36 (54–59) | 14.49 (13–17) | 41.13 (38–44) | 87.81 (86–90) | 59.67 (57–62) |

BMI, body mass index; COPD, Chronic Obstructive Pulmonary Disease.

**Table S4:** Overall and sex specific weighted unadjusted mean and 95 % CI of EQ-5D-3L index and EQ-VAS scores across behavioural risk factors and health conditions

|  |  | **EQ-5D index** |  |  | **EQ-VAS** |  |  |  |
| --- | --- | --- | --- | --- | --- | --- | --- | --- |
|  |  | *All* | *Male* | *Female* | *All* | *Male* | *Female* | |
| **Total (23 – 104 yr olds)** |  | 0.81 (0.81– 0.82) | 0.83 (0.83– 0.84) | 0.79 (0.79– 0.80) | 76.97 (76.69–77.24) | 78.02 (77.62– 78.42) | 75.96 (75.59–76.33) | |
| **Behavioural and metabolic risk factors** | |  |  |  |  |  |  | |
| BMI | < 18.5 | 0.73 (0.69–0.78) | 0.74 (0.64–0.84) | 0.73 (0.68–0.78) | 72.67 (69.59–75.76) | 71.97 (65.32–78.62) | 72.95 (69.55–76.36) | |
|  | 18.5 – 24.9 | 0.84 (0.83–0.84) | 0.85 (0.84–0.86) | 0.83 (0.82–0.83) | 79.00 (78.62–79.39) | 79.73 (79.10–80.35) | 78.45 (77.96–78.93) | |
|  | 25.0 – 29.9 | 0.81 (0.80–0.82) | 0.83 (0.83–0.84) | 0.78 (0.77–0.79) | 76.77 (76.34–77.21) | 78.15 (77.58–78.73) | 74.93 (74.26–75.60) | |
|  | 30.0 – 34.9 | 0.76 (0.75–0.77) | 0.79 (0.78–0.81) | 0.73 (0.72–0.75) | 73.02 (72.23–73.82) | 74.89 (73.84–75.94) | 70.98 (69.81–72.15) | |
|  | 35.0 – 39.9 | 0.72 (0.70–0.75) | 0.77 (0.74–0.80) | 0.69 (0.66–0.73) | 69.72 (68.09–71.35) | 71.70 (68.76–74.65) | 68.40 (66.48–70.31) | |
|  | ≥ 40.0 | 0.67 (0.63–0.72) | 0.73 (0.65–0.81) | 0.63 (0.58–0.68) | 66.33 (63.00–69.66) | 68.36 (61.95–74.78) | 64.78 (61.65–67.91) | |
| Smoking | Dailys moking | 0.75 (0.73–0.77) | 0.78 (0.75–0.80) | 0.73 (0.71–0.75) | 73.03 (71.84–74.23) | 74.19 (72.59–75.79) | 72.10 (70.40–73.80) | |
|  | Quit smoking | 0.79 (0.79–0.80) | 0.81 (0.80–0.82) | 0.78 (0.77–0.79) | 75.76 (75.27–76.25) | 76.16 (75.39–76.94) | 75.42 (74.79–76.05) | |
|  | Never | 0.82 (0.82–0.83) | 0.84 (0.84–0.85) | 0.80 (0.80–0.81) | 77.72 (77.38–78.05) | 78.85 (78.37–79.34) | 76.58 (76.12–77.03) | |
| Alcohol | Abstained | 0.74 (0.73–0.75) | 0.77 (0.74–0.79) | 0.73 (0.71–0.74) | 71.53 (70.59–72.48) | 73.46 (71.79–75.14) | 70.25 (69.14–71.36) | |
|  | Not hazardous | 0.83 (0.82–0.83) | 0.85 (0.84–0.85) | 0.81 (0.80–0.81) | 78.25 (77.97–78.54) | 79.18 (78.76–79.59) | 77.35 (76.96–77.75) | |
|  | Hazardous | 0.82 (0.81–0.83) | 0.82 (0.81–0.83) | 0.81 (0.80–0.82) | 77.07 (76.39–77.75) | 76.96 (76.01–77.90) | 77.21 (76.23–78.19) | |
| Sitting (hours/day) | <4 | 0.83 (0.82–0.84) | 0.85 (0.84–0.86) | 0.81 (0.80–0.82) | 79.29 (78.59–79.98) | 80.93 (79.85–82.01) | 77.97 (77.07–78.87) | |
|  | 04–09 | 0.82 (0.82–0.82) | 0.84 (0.84–0.85) | 0.80 (0.80–0.81) | 77.56 (77.23–77.88) | 78.82 (78.35–79.29) | 76.43 (75.98–76.88) | |
|  | 10+ | 0.78 (0.78–0.79) | 0.80 (0.79–0.82) | 0.76 (0.75–0.77) | 74.28 (73.66–74.90) | 75.06 (74.18–75.94) | 73.31 (72.45–74.18) | |
| Self-rated health | Very good | 0.94 (0.94–0.95) | 0.95 (0.94–0.95) | 0.94 (0.93–0.94) | 89.60 (89.27–89.92) | 89.81 (89.37–90.25) | 89.36 (88.89–89.83) | |
|  | Good | 0.85 (0.85–0.86) | 0.86 (0.86–0.87) | 0.84 (0.84–0.85) | 80.03 (79.78–80.28) | 80.20 (79.83–80.57) | 79.85 (79.51–80.19) | |
|  | Neither | 0.68 (0.68–0.69) | 0.70 (0.69–0.71) | 0.67 (0.66–0.68) | 64.20 (63.72–64.68) | 64.85 (64.10–65.59) | 63.70 (63.07–64.32) | |
|  | Bad | 0.40 (0.37–0.42) | 0.44 (0.40–0.48) | 0.36 (0.34–0.39) | 43.28 (42.10–44.47) | 43.99 (42.01–45.98) | 42.75 (41.32–44.17) | |
|  | Very bad | 0.07 (-0.01–0.15) | 0.05 (-0.10–0.19) | 0.08 (-0.01–0.17) | 28.77 (23.95–33.58) | 26.68 (17.25–36.12) | 30.38 (26.11–34.65) | |
| **Self-reported health conditions** | |  |  |  |  |  |  | |
| Neck pain | No | 0.84 (0.84–0.85) | 0.85 (0.85–0.86) | 0.83 (0.83–0.84) | 78.98 (78.70–79.26) | 79.44 (79.03–79.84) | 78.49 (78.10–78.88) | |
|  | Yes | 0.66 (0.65–0.67) | 0.68 (0.66–0.70) | 0.66 (0.64–0.67) | 67.56 (66.84–68.28) | 68.29 (66.95–69.63) | 67.17 (66.32–68.01) | |
| Low back pain | No | 0.85 (0.84–0.85) | 0.86 (0.85–0.86) | 0.83 (0.83–0.84) | 79.05 (78.78–79.32) | 79.70 (79.31–80.09) | 78.38 (78.01–78.76) | |
|  | Yes | 0.64 (0.63–0.65) | 0.66 (0.64–0.68) | 0.63 (0.62–0.65) | 66.16 (65.36–66.95) | 66.91 (65.49–68.33) | 65.67 (64.73–66.61) | |
| Headache/migraine | No | 0.83 (0.82–0.83) | 0.84 (0.84–0.85) | 0.81 (0.81–0.81) | 77.91 (77.65–78.18) | 78.65 (78.26–79.04) | 77.16 (76.80–77.52) | |
|  | Yes | 0.63 (0.61–0.65) | 0.60 (0.55–0.66) | 0.64 (0.62–0.66) | 63.64 (62.21–65.07) | 61.78 (58.54–65.02) | 64.34 (62.80–65.87) | |
| **Physician diagnosed conditions** | |  |  |  |  |  |  | |
| Diabetes | No | 0.82 (0.82–0.82) | 0.84 (0.84–0.85) | 0.80 (0.80–0.81) | 77.65 (77.37–77.94) | 78.79 (78.37–79.21) | 76.60 (76.22–76.98) | |
|  | Yes | 0.72 (0.71–0.74) | 0.75 (0.73–0.77) | 0.68 (0.66–0.70) | 68.76 (67.74–69.79) | 70.45 (69.07–71.83) | 66.43 (64.93–67.94) | |
| Angina pectoris | No | 0.82 (0.82–0.82) | 0.84 (0.83–0.84) | 0.80 (0.80–0.80) | 77.49 (77.21–77.76) | 78.64 (78.23–79.05) | 76.40 (76.04–76.77) | |
|  | Yes | 0.66 (0.63–0.69) | 0.70 (0.67–0.73) | 0.59 (0.54–0.64) | 63.87 (62.11–65.64) | 65.32 (63.27–67.36) | 61.10 (57.82–64.39) | |
| Heart failure | No | 0.82 (0.82–0.82) | 0.84 (0.84–0.85) | 0.80 (0.80–0.81) | 77.63 (77.36–77.91) | 78.78 (78.37–79.19) | 76.55 (76.18–76.92) | |
|  | Yes | 0.63 (0.61–0.65) | 0.66 (0.63–0.69) | 0.59 (0.55–0.63) | 61.63 (60.15–63.10) | 62.26 (60.41–64.12) | 60.71 (58.31–63.12) | |
| Hypertension | No | 0.83 (0.83–0.84) | 0.85 (0.84–0.86) | 0.82 (0.81–0.82) | 78.54 (78.23–78.86) | 79.59 (79.11–80.07) | 77.56 (77.14–77.98) | |
|  | Yes | 0.74 (0.74–0.75) | 0.77 (0.76–0.78) | 0.71 (0.70–0.72) | 71.62 (71.14–72.10) | 72.94 (72.27–73.60) | 70.26 (69.57–70.96) | |
| Asthma | No | 0.82 (0.82–0.82) | 0.84 (0.83–0.84) | 0.80 (0.80–0.81) | 77.51 (77.23–77.80) | 78.43 (78.01–78.85) | 76.61 (76.22–76.99) | |
|  | Yes | 0.75 (0.74–0.76) | 0.79 (0.77–0.81) | 0.72 (0.71–0.74) | 73.09 (72.17–74.01) | 74.83 (73.38–76.28) | 71.96 (70.78–73.13) | |
| COPD | No | 0.82 (0.81–0.82) | 0.84 (0.83–0.84) | 0.80 (0.80–0.80) | 77.41 (77.14–77.69) | 78.49 (78.08–78.89) | 76.38 (76.01–76.75) | |
|  | Yes | 0.64 (0.61–0.67) | 0.65 (0.62–0.69) | 0.63 (0.59–0.66) | 63.63 (61.94–65.32) | 62.08 (59.50–64.66) | 64.90 (62.68–67.12) | |
| Psoriasis | No | 0.82 (0.81–0.82) | 0.84 (0.83–0.84) | 0.80 (0.79–0.80) | 77.21 (76.93–77.50) | 78.29 (77.87–78.70) | 76.19 (75.81–76.57) | |
|  | Yes | 0.77 (0.75–0.78) | 0.79 (0.77–0.81) | 0.74 (0.72–0.77) | 74.51 (73.40–75.63) | 75.47 (73.82–77.12) | 73.50 (72.02–74.98) | |
| Hyperlipidemia | No | 0.82 (0.82–0.83) | 0.84 (0.84–0.85) | 0.81 (0.80–0.81) | 77.84 (77.55–78.13) | 79.03 (78.59–79.46) | 76.73 (76.34–77.12) | |
|  | Yes | 0.74 (0.73–0.75) | 0.76 (0.75–0.77) | 0.71 (0.69–0.73) | 71.23 (70.52–71.94) | 71.87 (70.90–72.84) | 70.46 (69.41–71.51) | |
| **Long-term illness** | No | 0.88 (0.87–0.88) | 0.89 (0.88–0.89) | 0.87 (0.87–0.87) | 81.63 (81.34–81.92) | 82.15 (81.73–82.57) | 81.10 (80.69–81.50) | |
|  | Yes | 0.69 (0.68–0.69) | 0.72 (0.71–0.73) | 0.66 (0.65–0.67) | 67.98 (67.49–68.47) | 69.30 (68.53–70.07) | 66.85 (66.22–67.48) | |
| **Multimorbidity** | 0 | 0.88 (0.88–0.89) | 0.89 (0.88–0.90) | 0.88 (0.87–0.88) | 81.82 (81.46–82.18) | 82.15 (81.62–82.68) | 81.45 (80.96–81.93) | |
|  | 1 | 0.81 (0.80–0.82) | 0.82 (0.81–0.83) | 0.80 (0.79–0.81) | 77.12 (76.60–77.64) | 77.72 (76.92–78.52) | 76.59 (75.91–77.27) | |
|  | 2 | 0.74 (0.73–0.74) | 0.77 (0.75–0.78) | 0.71 (0.70–0.73) | 71.93 (71.28–72.59) | 73.35 (72.45–74.26) | 70.85 (69.93–71.77) | |
|  | 3 | 0.68 (0.67–0.70) | 0.72 (0.69–0.75) | 0.65 (0.63–0.67) | 66.87 (65.80–67.94) | 68.49 (66.67–70.30) | 65.55 (64.28–66.81) | |
|  | 4+ | 0.57 (0.55–0.59) | 0.61 (0.59–0.64) | 0.54 (0.51–0.56) | 59.35 (58.22–60.49) | 60.70 (59.01–62.38) | 58.23 (56.68–59.77) | |
|  |  |  |  |  |  |  |  |  |

BMI, body mass index; COPD, Chronic Obstructive Pulmonary Disease;

**Table S5:** Differences in EQ-5D-3L index and EQ VAS scores across sociodemographic groups–risk factors and health conditions

|  |  | **EQ-5D index values** | |  | **EQ VAS*** |  |  |
| --- | --- | --- | --- | --- | --- | --- | --- |
|  |  | *Model 0* | *Model 1* | *Model 2* | *Model 0* | *Model 1* | *Model 2* |
| **Age** | 23–44 | ref | – – | – – | ref | – – | – – |
|  | 35–44 | 0.02 (0.00– 0.03) | – – | – – | 0.02 (0.01– 0.03) | – – | – – |
|  | 45–54 | 0.01 (-0.00– 0.02) | – – | – – | 0.02 (0.01– 0.03) | – – | – – |
|  | 55–64 | -0.03 (-0.05– -0.02) | – – | – – | -0.00 (-0.01– 0.01) | – – | – – |
|  | 65–84 | -0.05 (-0.07– -0.04) | – – | – – | -0.02 (-0.03– -0.01) | – – | – – |
|  | 85+ | -0.19 (-0.21– -0.17) | – – | – – | -0.15 (-0.16– -0.13) | – – | – – |
| **Sex** | Male | ref | ref | – – | ref | ref | – – |
|  | Female | -0.04 (-0.05– -0.03) | -0.04 (-0.04– -0.03) | – – | -0.02 (-0.03– -0.02) | -0.02 (-0.02– -0.01) | – – |
| **Income** | Q1 | -0.13 (-0.15– -0.12) | -0.12 (-0.14– -0.11) | – – | -0.11 (-0.12– -0.10) | -0.09 (-0.11– -0.08) | – – |
|  | Q2 | -0.08 (-0.09– -0.07) | -0.07 (-0.08– -0.06) | – – | -0.07 (-0.08– -0.06) | -0.06 (-0.07– -0.05) | – – |
|  | Q3 | -0.03 (-0.04– -0.03) | -0.04 (-0.05– -0.03) | – – | -0.03 (-0.04– -0.02) | -0.03 (-0.04– -0.02) | – – |
|  | Q4 | -0.01 (-0.02– -0.00) | -0.02 (-0.02– -0.01) | – – | -0.01 (-0.02– -0.00) | -0.01 (-0.02– -0.01) | – – |
|  | Q5 | ref | ref | – – | ref | ref | – – |
| **Education** | EducationL1 | -0.09 (-0.10– -0.08) | -0.05 (-0.07– -0.04) | – – | -0.06 (-0.07– -0.05) | -0.04 (-0.05– -0.03) | – – |
|  | EducationL2 | -0.04 (-0.05– -0.04) | -0.03 (-0.04– -0.02) | – – | -0.02 (-0.03– -0.02) | -0.02 (-0.03– -0.01) | – – |
|  | EducationL3 | ref | ref | – – | ref | ref | – – |
| **Country of birth** | Sweden | ref | ref | – – | ref | ref | – – |
|  | Nordic | -0.05 (-0.06– -0.04) | -0.02 (-0.03– -0.00) | – – | -0.03 (-0.04– -0.02) | -0.01 (-0.02– -0.00) | – – |
|  | EU28 | -0.02 (-0.04– -0.01) | -0.01 (-0.02– 0.01) | – – | 0.00 (-0.01– 0.01) | 0.01 (-0.00– 0.02) | – – |
|  | Other | -0.02 (-0.03– -0.01) | -0.02 (-0.03– -0.01) | – – | -0.01 (-0.02– -0.00) | -0.01 (-0.02– -0.01) | – – |
| **BMI status** | < 18.5 | -0.11 (-0.15– -0.06) | -0.10 (-0.14– -0.05) | -0.08 (-0.12– -0.04) | -0.06 (-0.09– -0.03) | -0.05 (-0.08– -0.02) | -0.04 (-0.07– -0.01) |
|  | 18.5 – 24.9 | ref | ref | ref | ref | ref | ref |
|  | 25.0 – 29.9 | -0.03 (-0.03– -0.02) | -0.02 (-0.03– -0.01) | -0.02 (-0.03– -0.01) | -0.02 (-0.03– -0.02) | -0.02 (-0.03– -0.02) | -0.02 (-0.03– -0.02) |
|  | 30.0 – 34.9 | -0.07 (-0.09– -0.06) | -0.07 (-0.08– -0.06) | -0.06 (-0.07– -0.05) | -0.06 (-0.07– -0.05) | -0.06 (-0.07– -0.05) | -0.05 (-0.06– -0.04) |
|  | 35.0 – 39.9 | -0.11 (-0.14– -0.09) | -0.11 (-0.14– -0.09) | -0.10 (-0.12– -0.07) | -0.09 (-0.11– -0.08) | -0.10 (-0.11– -0.08) | -0.09 (-0.10– -0.07) |
|  | ≥ 40.0 | -0.16 (-0.21– -0.12) | -0.17 (-0.21– -0.12) | -0.14 (-0.19– -0.10) | -0.13 (-0.16– -0.09) | -0.13 (-0.17– -0.10) | -0.12 (-0.15– -0.08) |
| **Smoking status** | Never | ref | ref | ref | ref | ref | ref |
|  | Quit smoking | -0.03 (-0.04– -0.02) | -0.02 (-0.03– -0.01) | -0.02 (-0.02– -0.01) | -0.02 (-0.03– -0.01) | -0.01 (-0.02– -0.01) | -0.01 (-0.02– -0.01) |
|  | Dailys moking | -0.07 (-0.09– -0.06) | -0.07 (-0.08– -0.05) | -0.04 (-0.06– -0.03) | -0.05 (-0.06– -0.03) | -0.05 (-0.06– -0.03) | -0.03 (-0.04– -0.02) |
| **Alcohol consumption** | Abstained | ref | ref | ref | ref | ref | ref |
|  | Not hazardous | 0.09 (0.07– 0.10) | 0.08 (0.07– 0.09) | 0.06 (0.04– 0.07) | 0.07 (0.06– 0.08) | 0.06 (0.05– 0.07) | 0.05 (0.04– 0.06) |
|  | Hazardous | 0.07 (0.06– 0.09) | 0.06 (0.04– 0.08) | 0.04 (0.02– 0.05) | 0.06 (0.04– 0.07) | 0.05 (0.03– 0.06) | 0.03 (0.02– 0.04) |
| **Sitting (hours/day)** | <04 hrs | ref | ref | ref | ref | ref | ref |
|  | 04–09 hrs | -0.01 (-0.02– -0.00) | -0.01 (-0.02– -0.00) | -0.03 (-0.04– -0.02) | -0.02 (-0.03– -0.01) | -0.02 (-0.03– -0.01) | -0.03 (-0.03– -0.02) |
|  | 10+ hrs | -0.05 (-0.06– -0.03) | -0.06 (-0.07– -0.05) | -0.08 (-0.09– -0.07) | -0.05 (-0.06– -0.04) | -0.06 (-0.07– -0.05) | -0.07 (-0.08– -0.06) |
| **Neck pain** | No | ref | ref | ref | ref | ref | ref |
|  | Yes | -0.18 (-0.19– -0.17) | -0.18 (-0.19– -0.17) | -0.17 (-0.18– -0.16) | -0.11 (-0.12– -0.11) | -0.11 (-0.12– -0.11) | -0.11 (-0.12– -0.10) |
| **Low back pain** | No | ref | ref | ref | ref | ref | ref |
|  | Yes | -0.20 (-0.21– -0.19) | -0.19 (-0.20– -0.18) | -0.18 (-0.19– -0.17) | -0.13 (-0.14– -0.12) | -0.12 (-0.13– -0.11) | -0.12 (-0.12– -0.11) |
| **Headache/migraine** | No | ref | ref | ref | ref | ref | ref |
|  | Yes | -0.20 (-0.22– -0.18) | -0.20 (-0.23– -0.18) | -0.19 (-0.21– -0.17) | -0.14 (-0.16– -0.13) | -0.15 (-0.16– -0.13) | -0.14 (-0.15– -0.12) |
| **Diabetes** | No | ref | ref | ref | ref | ref | ref |
|  | Yes | -0.10 (-0.11– -0.08) | -0.07 (-0.09– -0.06) | -0.06 (-0.08– -0.05) | -0.09 (-0.10– -0.08) | -0.07 (-0.09– -0.06) | -0.07 (-0.08– -0.06) |
| **Angina pectoris** | No | ref | ref | ref | ref | ref | ref |
|  | Yes | -0.16 (-0.19– -0.13) | -0.12 (-0.14– -0.09) | -0.11 (-0.14– -0.09) | -0.14 (-0.15– -0.12) | -0.11 (-0.13– -0.09) | -0.11 (-0.12– -0.09) |
| **Heart failure** | No | ref | ref | ref | ref | ref | ref |
|  | Yes | -0.19 (-0.21– -0.17) | -0.14 (-0.16– -0.12) | -0.14 (-0.16– -0.11) | -0.16 (-0.18– -0.15) | -0.12 (-0.14– -0.11) | -0.12 (-0.14– -0.11) |
| **Hypertension** | No | ref | ref | ref | ref | ref | ref |
|  | Yes | -0.09 (-0.10– -0.08) | -0.06 (-0.07– -0.05) | -0.06 (-0.07– -0.05) | -0.07 (-0.08– -0.06) | -0.06 (-0.07– -0.05) | -0.06 (-0.06– -0.05) |
| **Asthma** | No | ref | ref | ref | ref | ref | ref |
|  | Yes | -0.07 (-0.08– -0.06) | -0.07 (-0.09– -0.06) | -0.06 (-0.08– -0.05) | -0.04 (-0.05– -0.03) | -0.05 (-0.05– -0.04) | -0.04 (-0.05– -0.03) |
| **COPD** | No | ref | ref | ref | ref | ref | ref |
|  | Yes | -0.18 (-0.20– -0.15) | -0.14 (-0.17– -0.11) | -0.12 (-0.15– -0.10) | -0.14 (-0.15– -0.12) | -0.12 (-0.13– -0.10) | -0.10 (-0.12– -0.09) |
| **Psoriasis** | No | ref | ref | ref | ref | ref | ref |
|  | Yes | -0.05 (-0.07– -0.03) | -0.04 (-0.06– -0.03) | -0.04 (-0.06– -0.03) | -0.03 (-0.04– -0.02) | -0.02 (-0.04– -0.01) | -0.02 (-0.03– -0.01) |
| **Hyperlipidemia** | No | ref | ref | ref | ref | ref | ref |
|  | Yes | -0.09 (-0.10– -0.07) | -0.06 (-0.07– -0.05) | -0.06 (-0.07– -0.05) | -0.07 (-0.07– -0.06) | -0.05 (-0.06– -0.05) | -0.05 (-0.06– -0.04) |
| **Multimorbidity** | 0 diagnoses | ref | ref | ref | ref | ref | ref |
|  | 1 diagnoses | -0.07 (-0.08– -0.06) | -0.07 (-0.08– -0.07) | -0.07 (-0.08– -0.06) | -0.05 (-0.05– -0.04) | -0.05 (-0.06– -0.04) | -0.05 (-0.06– -0.04) |
|  | 2 diagnoses | -0.15 (-0.16– -0.14) | -0.15 (-0.16– -0.14) | -0.14 (-0.15– -0.13) | -0.10 (-0.11– -0.09) | -0.10 (-0.11– -0.10) | -0.10 (-0.11– -0.09) |
|  | 3 diagnoses | -0.20 (-0.22– -0.18) | -0.20 (-0.22– -0.19) | -0.19 (-0.21– -0.18) | -0.15 (-0.16– -0.14) | -0.16 (-0.17– -0.15) | -0.15 (-0.16– -0.14) |
|  | 4+ diagnoses | -0.31 (-0.33– -0.29) | -0.31 (-0.33– -0.29) | -0.30 (-0.32– -0.28) | -0.22 (-0.24– -0.21) | -0.23 (-0.25– -0.22) | -0.22 (-0.23– -0.21) |
| **Long-term illness** | No | ref | ref | ref | ref | ref | ref |
|  | Yes | -0.19 (-0.20– -0.18) | -0.19 (-0.19– -0.18) | -0.18 (-0.19– -0.17) | -0.14 (-0.14– -0.13) | -0.13 (-0.14– -0.13) | -0.13 (-0.13– -0.12) |
| **Self-rated health** | Very good | ref | ref | ref | ref | ref | ref |
|  | Good | -0.09 (-0.09– -0.08) | -0.09 (-0.09– -0.08) | -0.09 (-0.09– -0.08) | -0.10 (-0.10– -0.09) | -0.10 (-0.10– -0.09) | -0.10 (-0.10– -0.09) |
|  | Neither | -0.26 (-0.27– -0.25) | -0.25 (-0.26– -0.25) | -0.25 (-0.26– -0.24) | -0.25 (-0.26– -0.25) | -0.26 (-0.26– -0.25) | -0.25 (-0.26– -0.25) |
|  | Bad | -0.55 (-0.57– -0.52) | -0.54 (-0.56– -0.52) | -0.53 (-0.55– -0.51) | -0.46 (-0.48– -0.45) | -0.47 (-0.48– -0.45) | -0.46 (-0.47– -0.45) |
|  | Very bad | -0.88 (-0.96– -0.79) | -0.87 (-0.95– -0.79) | -0.85 (-0.94– -0.77) | -0.61 (-0.66– -0.56) | -0.61 (-0.66– -0.56) | -0.60 (-0.65– -0.55) |

Model 0–unadjusted; Model 1–adjusted for age; model 2–further adjusted for sex–income–education–country of birth.

*This is based on the data set with compete data on EQ VAS

BMI, body mass index; COPD, Chronic Obstructive Pulmonary Disease;

**Table S6:** Odds ratios for the probability of reporting less than perfect health (Part 1) and differences in EQ-5D-3L index (Part 2) comparing different sociodemographic groups–risk factors and health conditions

|  | | **Part 1 (OR of reporting less than perfect health)** | | | **Part 2 (differences in EQ-5D index)** | | |
| --- | --- | --- | --- | --- | --- | --- | --- |
|  |  | **Model 0** | **Model 1** | **Model 2** | **Model 0** | **Model 1** | **Model 2** |
| **Age** | 23–44 | ref | ref | ref | ref | ref | ref |
|  | 35–44 | 0.83 (0.73, 0.94) |  |  | 0.01 (-0.01, 0.02) |  |  |
|  | 45–54 | 0.84 (0.74, 0.95) |  |  | -0.01 (-0.02, 0.01) |  |  |
|  | 55–64 | 1.08 (0.95, 1.22) |  |  | -0.04 (-0.06, -0.03) |  |  |
|  | 65–84 | 1.41 (1.26, 1.59) |  |  | -0.05 (-0.06, -0.03) |  |  |
|  | 85+ | 4.06 (3.26, 5.06) |  |  | -0.14 (-0.17, -0.12) |  |  |
| **Sex** | Male | ref | ref | ref | ref | ref | ref |
|  | Female | 1.48 (1.38, 1.58) | 1.45 (1.35, 1.55) |  | -0.02 (-0.03, -0.01) | -0.02 (-0.03, -0.01) |  |
| **Income** | Q1 | 2.32 (2.04, 2.64) | 2.10 (1.84, 2.41) |  | -0.11 (-0.13, -0.10) | -0.11 (-0.13, -0.10) |  |
|  | Q2 | 1.88 (1.70, 2.09) | 1.73 (1.55, 1.93) |  | -0.06 (-0.07, -0.05) | -0.06 (-0.07, -0.05) |  |
|  | Q3 | 1.35 (1.22, 1.49) | 1.37 (1.24, 1.51) |  | -0.03 (-0.04, -0.01) | -0.03 (-0.04, -0.02) |  |
|  | Q4 | 1.20 (1.09, 1.32) | 1.23 (1.12, 1.36) |  | -0.00 (-0.01, 0.01) | -0.01 (-0.02, 0.00) |  |
|  | Q5 | ref | ref | ref | ref | ref | ref |
| **Education** | EducationL1 | 1.82 (1.66, 2.00) | 1.38 (1.24, 1.54) |  | -0.08 (-0.09, -0.07) | -0.05 (-0.06, -0.04) |  |
|  | EducationL2 | 1.33 (1.23, 1.43) | 1.24 (1.15, 1.35) |  | -0.04 (-0.05, -0.03) | -0.03 (-0.04, -0.02) |  |
|  | EducationL3 | ref | ref | ref | ref | ref | ref |
| **Country of birth** | Sweden | ref | ref | ref | ref | ref | ref |
|  | Nordic | 1.37 (1.21, 1.54) | 1.08 (0.96, 1.22) |  | -0.04 (-0.06, -0.03) | -0.02 (-0.03, -0.00) |  |
|  | EU28 | 1.12 (0.96, 1.31) | 1.02 (0.87, 1.20) |  | -0.02 (-0.04, -0.01) | -0.01 (-0.02, 0.01) |  |
|  | Other | 0.96 (0.87, 1.05) | 0.98 (0.89, 1.08) |  | -0.04 (-0.05, -0.03) | -0.04 (-0.05, -0.03) |  |
| **BMI status** | < 18.5 | 2.05 (1.47, 2.86) | 1.91 (1.36, 2.68) | 1.71 (1.21, 2.42) | -0.08 (-0.14, -0.03) | -0.08 (-0.13, -0.03) | -0.07 (-0.12, -0.02) |
|  | 18.5 – 24.9 | ref | ref | ref | ref | ref | ref |
|  | 25.0 – 29.9 | 1.25 (1.16, 1.34) | 1.22 (1.13, 1.32) | 1.26 (1.17, 1.36) | -0.02 (-0.03, -0.01) | -0.01 (-0.02, -0.01) | -0.01 (-0.02, 0.00) |
|  | 30.0 – 34.9 | 1.93 (1.72, 2.18) | 1.89 (1.68, 2.14) | 1.86 (1.65, 2.10) | -0.05 (-0.06, -0.03) | -0.04 (-0.05, -0.03) | -0.03 (-0.04, -0.01) |
|  | 35.0 – 39.9 | 2.59 (2.05, 3.27) | 2.64 (2.09, 3.33) | 2.44 (1.92, 3.11) | -0.07 (-0.10, -0.05) | -0.07 (-0.10, -0.05) | -0.06 (-0.09, -0.04) |
|  | ≥ 40.0 | 3.01 (1.85, 4.89) | 3.12 (1.91, 5.10) | 2.78 (1.71, 4.52) | -0.13 (-0.17, -0.08) | -0.13 (-0.17, -0.08) | -0.10 (-0.15, -0.06) |
| **Smoking status** | Never | ref | ref | ref | ref | ref | ref |
|  | Quit smoking | 1.40 (1.30, 1.51) | 1.31 (1.21, 1.42) | 1.26 (1.17, 1.37) | -0.01 (-0.02, -0.00) | -0.00 (-0.01, 0.01) | -0.00 (-0.01, 0.01) |
|  | Dailys moking | 1.70 (1.46, 1.97) | 1.65 (1.41, 1.91) | 1.45 (1.24, 1.69) | -0.05 (-0.07, -0.04) | -0.05 (-0.07, -0.03) | -0.03 (-0.05, -0.01) |
| **Alcohol** | Abstained | ref | ref | ref | ref | ref | ref |
|  | Not hazardous | 0.72 (0.65, 0.80) | 0.75 (0.68, 0.84) | 0.85 (0.76, 0.95) | 0.10 (0.08, 0.11) | 0.09 (0.07, 0.10) | 0.07 (0.05, 0.08) |
|  | Hazardous | 0.85 (0.74, 0.97) | 0.93 (0.81, 1.06) | 1.05 (0.91, 1.22) | 0.09 (0.08, 0.11) | 0.08 (0.06, 0.10) | 0.06 (0.04, 0.07) |
| **Sitting** | <04hrs | ref | ref | ref | ref | ref | ref |
|  | 04-09hrs | 1.15 (1.04, 1.26) | 1.16 (1.06, 1.28) | 1.26 (1.15, 1.39) | -0.00 (-0.01, 0.01) | -0.00 (-0.02, 0.01) | -0.02 (-0.03, -0.01) |
|  | 10hrs+ | 1.46 (1.30, 1.64) | 1.61 (1.43, 1.81) | 1.87 (1.65, 2.11) | -0.03 (-0.05, -0.02) | -0.05 (-0.06, -0.03) | -0.06 (-0.08, -0.05) |
| **Neck pain** | No | ref | ref | ref | ref | ref | ref |
|  | Yes | 6.57 (5.76, 7.50) | 6.73 (5.89, 7.69) | 6.34 (5.54, 7.25) | -0.10 (-0.12, -0.09) | -0.10 (-0.12, -0.09) | -0.10 (-0.11, -0.09) |
| **Low back pain** | No | ref | ref | ref | ref | ref | ref |
|  | Yes | 7.73 (6.64, 8.99) | 7.39 (6.35, 8.61) | 7.02 (6.03, 8.17) | -0.12 (-0.13, -0.11) | -0.12 (-0.13, -0.10) | -0.11 (-0.12, -0.10) |
| **Headache/migraine** | No | ref | ref | ref | ref | ref | ref |
|  | Yes | 6.17 (4.80, 7.92) | 6.55 (5.09, 8.42) | 5.93 (4.60, 7.65) | -0.13 (-0.15, -0.10) | -0.13 (-0.15, -0.11) | -0.12 (-0.15, -0.10) |
| **Diabetes** | No | ref | ref | ref | ref | ref | ref |
|  | Yes | 1.86 (1.63, 2.12) | 1.53 (1.34, 1.75) | 1.53 (1.34, 1.75) | -0.08 (-0.10, -0.06) | -0.06 (-0.08, -0.04) | -0.05 (-0.07, -0.03) |
| **Angina pectoris** | No | ref | ref | ref | ref | ref | ref |
|  | Yes | 2.57 (2.08, 3.16) | 1.93 (1.56, 2.38) | 2.01 (1.62, 2.49) | -0.13 (-0.16, -0.10) | -0.10 (-0.13, -0.07) | -0.09 (-0.12, -0.06) |
| **Heart failure** | No | ref | ref | ref | ref | ref | ref |
|  | Yes | 3.80 (3.07, 4.71) | 2.66 (2.14, 3.31) | 2.74 (2.20, 3.42) | -0.14 (-0.16, -0.12) | -0.10 (-0.13, -0.08) | -0.10 (-0.12, -0.08) |
| **Hypertension** | No | ref | ref | ref | ref | ref | ref |
|  | Yes | 2.06 (1.92, 2.21) | 1.78 (1.65, 1.92) | 1.78 (1.65, 1.92) | -0.06 (-0.07, -0.05) | -0.04 (-0.05, -0.03) | -0.03 (-0.04, -0.02) |
| **Asthma** | No | ref | ref | ref | ref | ref | ref |
|  | Yes | 1.75 (1.53, 1.99) | 1.77 (1.55, 2.02) | 1.69 (1.48, 1.92) | -0.05 (-0.06, -0.03) | -0.05 (-0.06, -0.03) | -0.04 (-0.06, -0.03) |
| **COPD** | No | ref | ref | ref | ref | ref | ref |
|  | Yes | 4.12 (3.16, 5.39) | 3.16 (2.42, 4.14) | 2.90 (2.21, 3.81) | -0.12 (-0.15, -0.09) | -0.09 (-0.12, -0.07) | -0.08 (-0.11, -0.06) |
| **Psoriasis** | No | ref | ref | ref | ref | ref | ref |
|  | Yes | 1.74 (1.45, 2.08) | 1.68 (1.40, 2.01) | 1.68 (1.40, 2.02) | -0.02 (-0.04, -0.00) | -0.01 (-0.03, 0.00) | -0.01 (-0.03, 0.00) |
| **Hyperlipidemia** | No | ref | ref | ref | ref | ref | ref |
|  | Yes | 1.99 (1.81, 2.19) | 1.68 (1.52, 1.85) | 1.71 (1.54, 1.88) | -0.06 (-0.07, -0.05) | -0.04 (-0.05, -0.02) | -0.03 (-0.04, -0.02) |
| **Multimorbidity** | 0 diagnose | ref | ref | ref | ref | ref | ref |
|  | 1 diagnose | 2.24 (2.05, 2.43) | 2.32 (2.13, 2.53) | 2.29 (2.10, 2.50) | -0.04 (-0.05, -0.03) | -0.04 (-0.05, -0.03) | -0.03 (-0.04, -0.03) |
|  | 2 diagnoses | 4.42 (3.98, 4.90) | 4.72 (4.24, 5.24) | 4.53 (4.07, 5.04) | -0.09 (-0.10, -0.08) | -0.09 (-0.10, -0.07) | -0.08 (-0.09, -0.07) |
|  | 3 diagnoses | 6.92 (5.94, 8.07) | 7.55 (6.46, 8.82) | 7.26 (6.21, 8.49) | -0.13 (-0.14, -0.11) | -0.13 (-0.15, -0.11) | -0.12 (-0.14, -0.10) |
|  | 4+ diagnoses | 13.53 (10.73, 17.05) | 14.94 (11.80, 18.93) | 14.11 (11.11, 17.90) | -0.22 (-0.24, -0.20) | -0.22 (-0.24, -0.20) | -0.20 (-0.22, -0.19) |
| **Long lasting illness** | No | ref | ref | ref | ref | ref | ref |
|  | Yes | 5.68 (5.21, 6.20) | 5.53 (5.06, 6.05) | 5.40 (4.94, 5.91) | -0.13 (-0.14, -0.12) | -0.12 (-0.13, -0.12) | -0.12 (-0.13, -0.11) |
| **Self-rated health** | Very good | ref | ref | ref | ref | ref | ref |
|  | Good | 4.44 (4.05, 4.88) | 4.51 (4.10, 4.95) | 4.50 (4.09, 4.96) | -0.02 (-0.03, -0.02) | -0.02 (-0.03, -0.02) | -0.02 (-0.03, -0.01) |
|  | Neither | 40.13 (34.32, 46.91) | 40.62 (34.66, 47.60) | 40.04 (34.12, 46.98) | -0.13 (-0.14, -0.12) | -0.13 (-0.14, -0.11) | -0.12 (-0.13, -0.11) |
|  | Bad | 557.80 (225.62, 1379.07) | 567.86 (229.87, 1402.81) | 551.56 (222.96, 1364.46) | -0.40 (-0.42, -0.38) | -0.39 (-0.42, -0.37) | -0.38 (-0.41, -0.36) |
|  | Very bad | 551.76 (134.91, 2256.55) | 557.26 (136.55, 2274.15) | 542.52 (132.54, 2220.62) | -0.73 (-0.81, -0.65) | -0.72 (-0.81, -0.64) | -0.71 (-0.79, -0.62) |
|  |  |  |  |  |  |  |  |

Model 0–unadjusted; Model 1–adjusted for age; model 2–further adjusted for sex–income–education–country of birth.

BMI, body mass index; COPD, Chronic Obstructive Pulmonary Disease;

**Table S7**: results from sensitivity analysis comparing the mean (sd) of EQ-5D index values and EQ VAS scores–and prevalence of reported problems in each dimension between 2014 and 2021.

| **Age** | **Wave** | **EQ-5D index,**  **mean (sd)** | **EQ VAS,**  **mean (sd)** | **Mobility, %** | **Self-care, %** | **Usua activity, %** | **Pain/discomfort, %** | **Anxiety/depression, %** |
| --- | --- | --- | --- | --- | --- | --- | --- | --- |
| **23–29** | 2014, n=1439 | 0.85 (0.18) | 78.99 (16.23) | 3.54 | 0.7 | 8.71 | 31.63 | 44.34 |
|  | 2021, n=680 | 0.83 (0.20) | 76.49 (15.78) | 3.16 | 1.03 | 10.11 | 29.59 | 55.6 |
|  | **p-value^*^** | 0.046 | <0.001 | 0.7 | 0.5 | 0.4 | 0.4 | <0.001 |
| **30–34** | 2014, n=1292 | 0.87 (0.19) | 79.65 (16.95) | 2.51 | 0.78 | 6.92 | 28.49 | 38.12 |
|  | 2021, n=1236 | 0.83 (0.20) | 77.98 (15.02) | 4.79 | 0.8 | 9.03 | 34.64 | 49.25 |
|  | **p-value** | <0.001 | <0.001 | 0.013 | >0.9 | 0.09 | 0.004 | <0.001 |
| **35–39** | 2014, n=1475 | 0.86 (0.20) | 79.81 (16.31) | 5.02 | 0.94 | 6.25 | 33.49 | 34.08 |
|  | 2021, n=1844 | 0.85 (0.18) | 78.86 (14.51) | 4.92 | 0.75 | 7.32 | 33.57 | 43.78 |
|  | **p-value** | 0.007 | 0.002 | >0.9 | 0.6 | 0.3 | >0.9 | <0.001 |
| **40–44** | 2014, n=1819 | 0.86 (0.20) | 79.80 (15.91) | 5.22 | 1.5 | 7.62 | 36.54 | 32.54 |
|  | 2021, n=3063 | 0.85 (0.19) | 79.11 (14.59) | 5.31 | 0.84 | 7.53 | 38.84 | 39.33 |
|  | **p-value** | 0.009 | 0.012 | >0.9 | 0.11 | >0.9 | 0.2 | <0.001 |
| **45–49** | 2014, n=1727 | 0.84 (0.21) | 79.57 (16.81) | 7.31 | 1.29 | 7.36 | 43.13 | 30.18 |
|  | 2021, n=4095 | 0.84 (0.19) | 79.00 (14.90) | 7.5 | 1.25 | 7.9 | 41.56 | 36.47 |
|  | **p-value** | 0.4 | 0.005 | 0.9 | >0.9 | 0.6 | 0.4 | <0.001 |
| **50–54** | 2014, n=1919 | 0.82 (0.22) | 78.44 (19.10) | 9.74 | 2.5 | 10.61 | 47.8 | 33.37 |
|  | 2021, n=5012 | 0.83 (0.20) | 79.32 (15.31) | 10.34 | 2.1 | 8.63 | 47.82 | 33.77 |
|  | **p-value** | >0.9 | 0.2 | 0.6 | 0.5 | 0.062 | >0.9 | 0.8 |
| **55–59** | 2014, n=1631 | 0.79 (0.25) | 76.74 (19.47) | 16.74 | 4.24 | 13.3 | 51.86 | 37.2 |
|  | 2021, n=5651 | 0.80 (0.24) | 77.12 (17.63) | 15.23 | 3.52 | 11.62 | 52.9 | 35.72 |
|  | **p-value** | 0.6 | 0.5 | 0.3 | 0.4 | 0.2 | 0.6 | 0.4 |
| **60–64** | 2014, n=1764 | 0.79 (0.24) | 77.68 (19.12) | 16.67 | 3.41 | 13.51 | 56.87 | 34.03 |
|  | 2021, n=5575 | 0.80 (0.22) | 77.43 (16.89) | 18.77 | 2.77 | 12.77 | 57.3 | 33.15 |
|  | **p-value** | >0.9 | 0.019 | 0.15 | 0.3 | 0.6 | 0.8 | 0.6 |
| **65–69** | 2014, n=1947 | 0.81 (0.23) | 78.55 (18.65) | 19.04 | 3.27 | 10.57 | 55.16 | 29.39 |
|  | 2021, n=5735 | 0.80 (0.22) | 77.25 (18.16) | 22 | 2.95 | 12.09 | 57.54 | 31.39 |
|  | **p-value** | 0.13 | 0.003 | 0.05 | 0.6 | 0.2 | 0.2 | 0.2 |
| **70–74** | 2014, n=1785 | 0.81 (0.20) | 77.08 (18.83) | 20.96 | 2.88 | 9.94 | 56.87 | 27.23 |
|  | 2021, n=6545 | 0.79 (0.22) | 76.26 (17.32) | 26.15 | 4.15 | 12.78 | 61.73 | 30.71 |
|  | **p-value** | <0.001 | 0.007 | <0.001 | 0.068 | 0.015 | 0.005 | 0.032 |
| **75–79** | 2014, n=1072 | 0.77 (0.23) | 73.96 (19.90) | 30.27 | 6.7 | 15.73 | 66.34 | 31.72 |
|  | 2021, n=6854 | 0.77 (0.22) | 74.02 (18.14) | 32.19 | 5.33 | 17.39 | 64.66 | 30.44 |
|  | **p-value** | >0.9 | 0.4 | 0.3 | 0.2 | 0.3 | 0.4 | 0.5 |
| **80–84** | 2014, n=684 | 0.70 (0.27) | 66.28 (23.48) | 46.24 | 7.96 | 26.48 | 72.28 | 35.89 |
|  | 2021, n=4050 | 0.74 (0.25) | 70.45 (19.66) | 43.91 | 7.32 | 23.12 | 69.24 | 32.69 |
|  | **p-value** | 0.081 | 0.016 | 0.4 | 0.7 | 0.15 | 0.2 | 0.2 |
| **85–89** | 2014, n=397 | 0.64 (0.32) | 61.62 (24.36) | 62.55 | 19.25 | 39.81 | 75.35 | 42.56 |
|  | 2021, n=1718 | 0.66 (0.27) | 64.55 (20.49) | 61.04 | 12.72 | 37.14 | 77.45 | 41.73 |
|  | **p-value** | 0.7 | 0.4 | 0.7 | 0.017 | 0.5 | 0.5 | 0.8 |
| **90+** | 2014, n=230 | 0.54 (0.36) | 52.18 (25.83) | 76.94 | 33.16 | 55.71 | 81.32 | 47.46 |
|  | 2021, n=852 | 0.59 (0.33) | 59.04 (21.76) | 74.64 | 29.21 | 47.42 | 79.64 | 39.82 |
|  | **p-value** | 0.2 | 0.004 | 0.6 | 0.4 | 0.076 | 0.6 | 0.1 |

*^*^*Design-based Kruskal Wallis test; Pearson's X^2: Rao & Scott adjustment


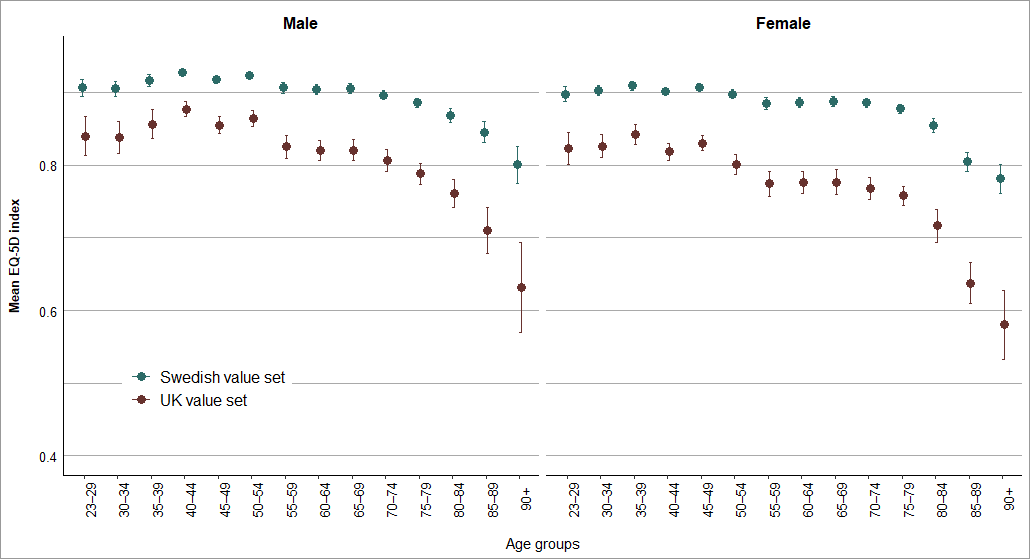


**Figure S1.** Sensitivity analyses results comparing the UK (used in the main analysis) and Swedish experience-based value sets.

Table S8. Mean and 95% CI of EQ-5D index values based on the UK and Swedish experience-based value sets.

|  | **UK value set** |  | **Swedish value set** |  |  |
| --- | --- | --- | --- | --- | --- |
| **Age** | **Male** | **Female** | **Male** | **Female** |  |
| 23–29 | 0.84 (0.81–0.87) | 0.82 (0.80–0.85) | 0.91 (0.89–0.92) | 0.90 (0.89–0.91) |  |
| 30–34 | 0.84 (0.82–0.86) | 0.83 (0.81–0.84) | 0.91 (0.90–0.92) | 0.90 (0.90–0.91) |  |
| 35–39 | 0.86 (0.84–0.88) | 0.84 (0.83–0.86) | 0.92 (0.91–0.93) | 0.91 (0.90–0.92) |  |
| 40–44 | 0.88 (0.87–0.89) | 0.82 (0.81–0.83) | 0.93 (0.92–0.93) | 0.90 (0.90–0.91) |  |
| 45–49 | 0.86 (0.84–0.87) | 0.83 (0.82–0.84) | 0.92 (0.91–0.92) | 0.91 (0.90–0.91) |  |
| 50–54 | 0.86 (0.85–0.88) | 0.80 (0.79–0.82) | 0.92 (0.92–0.93) | 0.90 (0.89–0.90) |  |
| 55–59 | 0.83 (0.81–0.84) | 0.77 (0.76–0.79) | 0.91 (0.90–0.91) | 0.89 (0.88–0.89) |  |
| 60–64 | 0.82 (0.81–0.83) | 0.78 (0.76–0.79) | 0.90 (0.90–0.91) | 0.89 (0.88–0.89) |  |
| 65–69 | 0.82 (0.81–0.84) | 0.78 (0.76–0.79) | 0.91 (0.90–0.91) | 0.89 (0.88–0.90) |  |
| 70–74 | 0.81 (0.79–0.82) | 0.77 (0.75–0.78) | 0.90 (0.89–0.90) | 0.89 (0.88–0.89) |  |
| 75–79 | 0.79 (0.77–0.80) | 0.76 (0.74–0.77) | 0.89 (0.88–0.89) | 0.88 (0.87–0.88) |  |
| 80–84 | 0.76 (0.74–0.78) | 0.72 (0.69–0.74) | 0.87 (0.86–0.88) | 0.85 (0.84–0.86) |  |
| 85–89 | 0.71 (0.68–0.74) | 0.64 (0.61–0.67) | 0.85 (0.83–0.86) | 0.80 (0.79–0.82) |  |
| 90+ | 0.63 (0.57–0.69) | 0.58 (0.53–0.63) | 0.80 (0.77–0.83) | 0.78 (0.76–0.80) |  |
